# Systemic risk prevention policies targeting systemically important banks: Does clustering pattern matter?

**DOI:** 10.1371/journal.pone.0284861

**Published:** 2023-04-27

**Authors:** Bo Zhu, Xin Hu, Yuanyue Deng, Renda Lin

**Affiliations:** School of Finance, Southwestern University of Finance and Economics, Chengdu, China; Alexandru Ioan Cuza University of Iasi, ROMANIA

## Abstract

It is well known that strengthening the soundness of individual banks that are too large or too interconnected is essential for maintaining financial stability. The clustering among homogeneous banks may also cause financial fragility but has received less attention. This paper discusses the policy improvement for preventing systemic risk from the perspective of the clustering pattern of systemically important banks (SIBs) based on a network optimization model. The results show that the clustering pattern of SIBs is closely related to systemic risk contagion. Remarkably, networks with fewer connections among SIBs exhibit lower systemic risk than those featuring apparent clustering of SIBs. The potential mechanism is that the systemic vulnerability of small and medium-sized banks is greatly reduced in the disassortative networks. The proposed tools based on this—inter-SIBs exposure limits and pairwise capital requirements—can guide network optimization and significantly reduce systemic risk. Furthermore, combining existing capital surcharges for SIBs (focusing on the stability of individual SIBs) and proposed network-based tools (focusing on the cluster structure of the network) will be an effective way to enhance financial stability over existing policies.

## 1. Introduction

After the global financial crisis in 2008, systemic risk arising from the contagion among financial institutions has attracted much attention. In recent years, a series of extreme events in China, such as the “money shortage” in 2013 and the “stock market crash” in 2015, have demonstrated that the complex network of credit relationships connecting banks’ balance sheets can exacerbate risk contagion under extreme shocks and even trigger a systemic crisis. Although China has not experienced a systemic financial crisis, the financial system’s stability has been successively affected by shocks such as the “major credit risk event of Baoshang Bank” and the “restructuring of Jinzhou Bank due to liquidity problems” in 2019. Therefore, identifying the interlinkages among financial institutions and preventing cross-sectional systemic risk contagion is of great theoretical and practical significance for enhancing financial stability and ensuring high-quality economic development.

**Fig 1:**
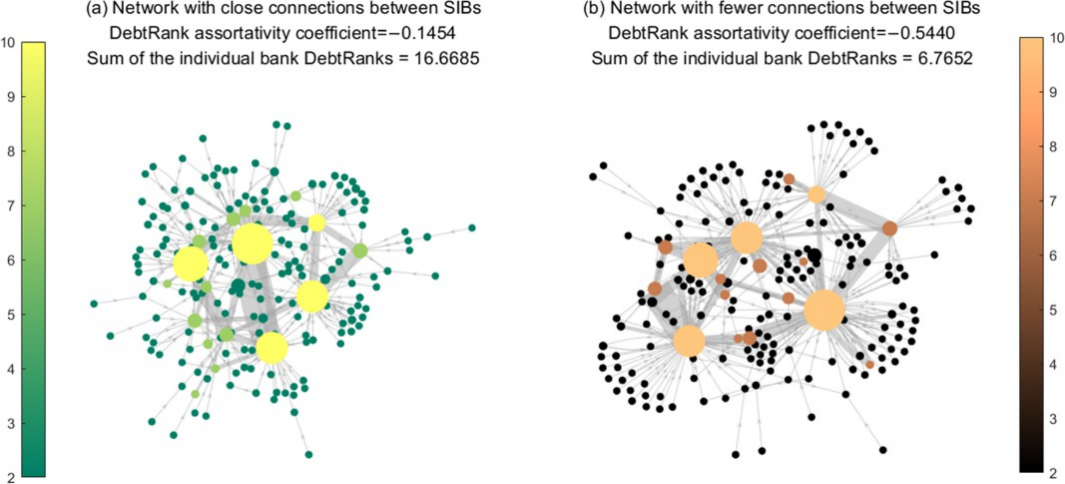
The systemic vulnerability in networks with different connection tendencies of SIBs. The interbank network in **Fig 1(a)** is estimated by the minimum density method based on the data of 188 banks’ interbank assets and interbank liability in 2019. In **Fig 1(b)**, the SIBs’ cluster patterns are restructured without changing the economic conditions of **Fig 1(a)**, such as total interbank assets/liabilities and capital buffer. The nodes’ size is positively related to banks’ equity in the figure. The amount of interbank lending/borrowing determines the thickness of the gray lines linking the nodes. Nodes with light to dark colors correspond to large-scale state-owned, joint-stock, and other banks, respectively. The DebtRank assortativity coefficient indicates banks’ tendency to connect with others with a similar DebtRank level.

In the increasingly uncertain external environment, there is a great tendency to cluster among homogeneous banks, generating apparent community/cluster structure in interbank networks. Particularly, systemically important banks (SIBs) always have close business connections [1–3]. Taking the Chinese market as an example, large state-owned commercial banks usually tend to establish business ties with banks of the same type (due to factors such as historical business relationships, similar business models, and easy access to liquidity) rather than with other small- and medium-sized banks. Under exogenous shocks, such a cluster structure may cause a more significant crisis because a core with close connections exacerbates contagion, as shown in Fig 1(a) and 1(b). In summary, the clustering pattern of SIBs, which refers to the propensity of nodes to form clusters, can alter the network configuration and impact risk diffusion mechanisms both within and between clusters. Consequently, it is crucial to pay close attention to this phenomenon.

**Fig 2:**
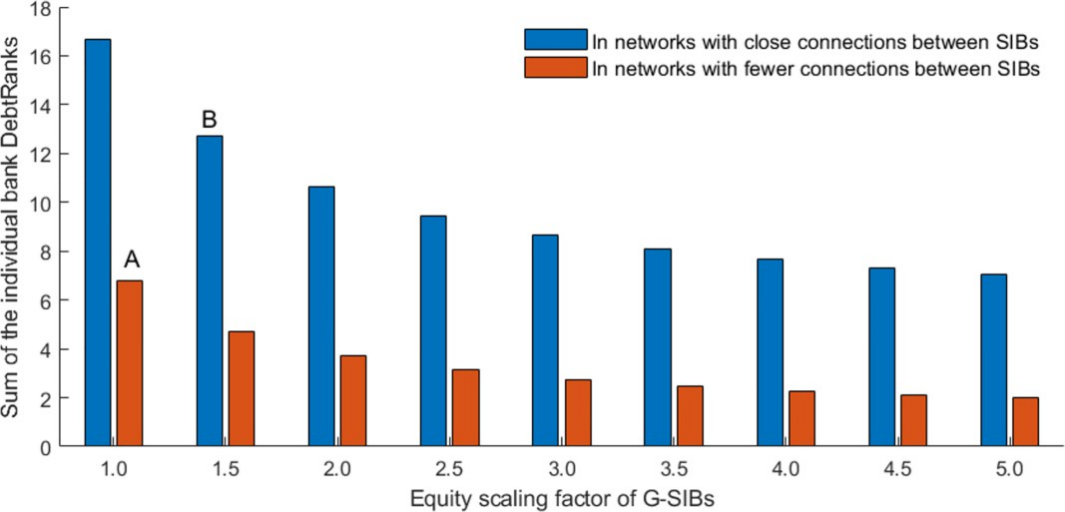
Systemic vulnerability in different networks and with different capital surcharges for SIBs. With the implementation of capital surcharges for G-SIBs, banks’ equity increases, but the interlinkages remain the same in Fig 1(a) and 1(b).

In order to limit systemic risk contagion in financial networks, regulators take the “too-big-to-fail” and “too-interconnected-to-fail” problems seriously and introduce many policies, such as the capital/leverage surcharges for systemically important banks (SIBs) proposed in Basel III. The identification of SIBs focuses on the “aggregate interconnectedness” of individual banks, and existing regulatory policies aim to enhance the stability of individual SIBs [4]. Nevertheless, the group interlinkages among SIBs described by their connection tendency and the resulting cluster structure of the interbank network are ignored. As a result, existing policies may be insufficient for regulating systemic risk [5, 6]. For instance, after implementing capital surcharges for SIBs, systemic vulnerability greatly increases rather than decreases due to the changes in SIBs’ connection preference, as shown in Fig 2 from point A to point B. Therefore, exploring policy tools for systemic risk prevention from SIBs’ clustering pattern perspective is particularly interesting.

Motivated by those problems, this paper aims to discuss the policy improvement for systemic risk prevention based on the clustering pattern of SIBs in the Chinese banking system. Our work includes three steps. First, based on Bluhm [7] and Diem et al. [8], this paper builds a banking network model embedded with a network optimization procedure to describe the network formulation process and risk contagion mechanism using the annual asset and liability data of four types of commercial banks in the Chinese market from 2010–2019. Second, the influences of SIBs’ connection tendency on systemic risk are analyzed. We estimate the optimized networks with a minimum loss in interbank assets by rearranging interlinkages without changing initial economic conditions. Third, depending on the cluster characteristics of optimized networks, we introduce two policy tools to guide the network toward the optimal design and test their effectiveness in the banking network model.

This paper examines the Chinese banking system for two primary reasons. Firstly, China is the world’s second-largest economy, with significant foreign exchange reserves and substantial cross-border capital flows. Thus, regulating systemic risk in China’s financial markets can significantly impact global financial markets. Secondly, Chinese commercial banks are largely homogeneous, with similar business structures, operating models, and financial products. The clustering pattern among SIBs could pose a significant source of systemic risk since they are usually closely related. Therefore, it is a typical example of investigating systemic risk in the Chinese banking system. Our study not only offers insights for financial authorities and policymakers in China but also sheds light on preventing systemic risk in the interconnected networks of global financial institutions.

The research contributes to the existing literature in three ways. First, based on the available data for 50 banks in the Chinese market, our study offers new insights for maintaining financial stability that regulators should take the group trading pattern of SIBs into account, in addition to the regulatory policies for the business behavior of individual SIBs. Second, this paper, for the first time, proposes the tools of inter-SIBs exposure limits and pairwise capital requirements to improve network stability. The research enriches the study on the improvement the systemic risk prevention policies. Third, this paper modifies the network optimization method of Diem et al. [8] by extending the systemic risk measurement to capture the reinforcement of the multiple risk transmission channels, thus offering a reference for the policy design in complex networks in practice.

This paper proceeds as follows. Section 2 reviews the related literature. Section 3 introduces the banking network model with an optimization procedure. Section 4 briefly discusses the data and details the optimization results. Section 5 proposes two tools that can lead the network to its optimal structure and tests their effectiveness in the banking network model. Finally, we summarize our findings in Section 6.

## 2. Related literature

Systemic risk is the risk of the collapse of an entire system or market [9]. It arises from the interconnectedness and institutions’ interdependence, which can lead to cascading failures and a collapse of the entire system or market when one or more individuals fail. The frequent crisis events in the past 20 years have attracted global countries’ attention to preventing systemic risk and improving regulatory policies. Our research mainly correlates to the following three aspects of literature about systemic risk prevention.

First, the individual SIBs’ stability has long been the focus of supervision. The “too-big-to-fail” [10, 11] and “too-interconnected-to-fail” [12, 13] views hold that the failure of financial institutions that are very large or are closely interconnected with others could pose a significant threat to financial stability. Those studies emphasize the importance of strengthening the soundness of individual institutions that are systemically important to ensure the financial system’s stability [4, 14]. However, in formulating systemic risk prevention policies, less attention has been paid to the network structure that is highly relevant to systemic risk contagion [15–19]. In particular, it is not yet known how the network configuration described by SIBs’ connection tendency will be incorporated into the regulatory system.

Second, studies assessing systemic risk based on bank networks highlight the multiple risk contagion channels, such as the risk contagion arising from exposure to the counterparty [20] and exposure through holding common assets [21]. However, research on the network optimization model only considers the single risk contagion channel and ignores the reinforcement of the multiple risk transmission channels. For example, Diem et al. [8] show the considerable potential to reduce systemic risk by about 70% by rearranging the interbank linkages in the Austrian interbank market. Pichler et al. [22] find reductions in systemic risk of around 50% by rearranging the government bond portfolios of the 49 European banks. Zedda and Sbaraglia [19] and Krause et al. [23] use Monte Carlo simulations to generate a series of networks and then explore the relationships between system stability and network structure.

Third, the network-based policy tools, which attach importance to the joint risk performance of many banks, have also received much attention. Poledna and Thurner [24] and Leduc and Thurner [25] propose a tool, systemic risk tax (SRT), to encourage banks to select counterparties that create less pressure on the system. Poledna et al. [5] then show that capital surcharges for G-SIBs introduced by Basel III are far less effective than SRT in controlling systemic risk. Coen and Coen [26] propose two tools to improve efficiency: caps on aggregate exposure and pairwise capital requirements based on the contagion intensity of each link. However, those policies are difficult to be applied in practice. Each transaction’s marginal systemic risk contribution and the contagion intensity of each link will change with each transaction, and getting information on each transaction is costly.

In summary, existing studies on systemic risk mainly focus on individual SIBs’ stability, and relatively little research has been conducted on the cluster structure of the network described by the trading pattern of SIBs. The kind of cluster patterns of SIBs that most resists risk contagion is also unclear in the networks with multiple risk contagion channels. Furthermore, how to guide the network toward the optimal design in practice still needs further exploration. This paper aims to answer these issues and enrich the existing literature on preventing systemic risk.

## 3. Banking network model

**Fig 3:**
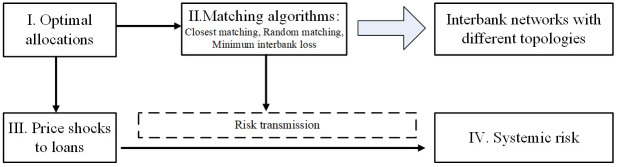
Overview of the banking network model.

Network models have been widely used to study the role of network structure in generating systemic risk and assess the risk transmission in the financial system [e.g., 7, 27–32]. Among them, the micro-founded network model of Bluhm [7] describes the formation of financial networks, the propagation of shocks, and the emergence of systemic risk. Based on Bluhm’s [7] framework, we further embed a network optimization procedure in the network formation process. It enables us to reveal the influences of network configuration described by SIBs’ connection tendency on the overall systemic risk and to offer helpful suggestions for policy improvement. Our model also differs from Bluhm’s [7] model by giving the deposit data exogenously and by ignoring the persistence of liquidity shocks. The overview of the banking network model is shown in Fig 3.

### 3.1 Banks’ allocations to maximize the expected profit

Table 1 shows a simplified bank balance sheet and the abbreviations for its components. Given *n* banks in the financial system *N*, they are assumed to choose their balance-sheet positions that maximize their expected profit in an environment with regulatory constraints, which can be expressed as

$$\underset{\left\{c_{i},bl_{i},bb_{i},ml_{i}\right\}}{\text{max}}\;E(\pi_{i})=bl_{i}\cdot r_{b}+P\cdot ml_{i}\cdot r_{m}(1-\frac{loans_{i}}{BS_{i}})-bb_{i}\cdot r_{b}\cdot \left(\frac{1}{1-LGD\cdot prob_{i}}\right)$$ (1)

s.t.

$$c_{i}\geq (h+{ο}_{i})\cdot d_{i}$$ (2)

$$bl_{i}\geq \varphi \cdot d_{i},bb_{i}\geq \varphi \cdot d_{i}$$ (3)

$$c_{i}+P\cdot ml_{i}+bl_{i}-d_{i}-bb_{i}\geq (\chi_{1}\cdot P\cdot ml_{i}+\chi_{2}\cdot bl_{i})\cdot (\gamma^{*}+\tau_{i})$$ (4)

$$c_{i}+P\cdot ml_{i}+bl_{i}-bb_{i}=e_{i}+d_{i}$$ (5)

$$c_{i}\geq 0,bl_{i}\geq 0,ml_{i}\geq 0,bb_{i}\geq 0$$ (6)

*r*_*b*_, *P*, *r*_*m*_, *BS*_*i*_, *LGD*, and *prob*_*i*_ denote the interbank market rate, the price of loans, the return of loans, banks’ branch size, the loss given default, and the expected probability of default, respectively. The interest rate of interbank borrowing is *r*_*b*_ (1/(1 − *LGD prob*_*i*_)) because borrowers are risky. Here, we delete the interest payments to depositors because banks’ deposits are given exogenously.

**Table 1:** The simplified balance sheet of bank *i*.

Assets	Liabilities and equity
Liquid assets (*c*_*i*_)	Deposits (*d*_*i*_)
Interbank lending (*bl*_*i*_)	Interbank borrowing (*bb*_*i*_)
Loans (*ml*_*i*_)	Equity (*e*_*i*_)

Eq (2) indicates the reserve requirements; *h* and *o*_*i*_ denote the required and excess reserve requirement ratio, respectively. Eq (3) indicates that the interbank funds are not less than *φ d*_i_, where *φ* is given exogenously. When banks face idiosyncratic liquidity shocks caused by the behaviors of retail depositors, they usually use unsecured interbank funds to hedge against short-term liquidity shortfalls [33]. So, the bank *i*’s interbank assets and liabilities can at least cover its liquidity demand. The capital requirements that bank *i* needs to meet are shown in Eq (4). *χ*_1_ and *χ*_2_ are the capital weights on loans and interbank lending, respectively. γ* is the minimum capital adequacy requirement ratio. *τ*_*i*_ denotes the capital buffer ratio. The accounting equation holds all the times as shown in Eq (5). Finally, Eq (6) indicates that all elements in the banks’ balance sheet are non-negative.

We use linear programming to obtain the solutions to bank balance-sheet positions {*c*_*i*_, *bl*_*i*_, *bb*_*i*_, *ml*_*i*_}. In the model, the market price of the loan portfolios (*P*) is later determined by the centralized tâtonnement process. Other parameters are given exogenously, as shown in Table 2. The equity (*e*_*i*_), deposit (*d*_*i*_), and individual banks’ reserve and capital preferences (*τ*_*i*_ and *o*_*i*_) determine their heterogeneity.

**Table 2:** Model parameters.

Symbol	Description	Value	Sources
*n*	Number of banks	50	Author selection
*e*_*i*_	Equity	Top 50 banks’ equity	Based on real data
*d*_*i*_	Deposit	Top 50 banks’ deposit	Based on real data
*r*_*m*_	Return on loans	8%	Bluhm [7]
*r*_*b*_	Interbank market rate	2.31%	Based on real data
*h*	Statutory deposit reserve ratio	13%	Based on real data
*o*_*i*_	Excess deposit reserve ratio	1%–2%	Based on real data
γ*	Capital adequacy requirement ratio	8%	Basel III capital requirements
*τ*_*i*_	Capital buffer requirement ratio	1%–2.5%	Based on real data
*χ*_1_	Capital weight on loans	1	Administrative Measures for the Capital of Commercial Banks of China (2012)
*χ*_2_	Capital weight on interbank credits	0.2	
*φ*_*i*_	Excess interbank funds	0.1169	Calculation based on real data (Total interbank assets/Total deposits)
Ψ	Shock to loan price	| N (0.04, 0.01) |	Bluhm [7]
*P*	Initial loan price	1	Bluhm [7]
*prob*_*i*_	Expected default probability	0.005	Select values in an appropriate range
*LGD*	Loss given default	0.5	Select values in an appropriate range

### 3.2 Network formation mechanism

Given the optimal allocations (**bl**, **bb**), we then estimate the interbank network configuration, that is, how the interbank funds are matched. This paper uses several alternative algorithms to generate different interbank bilateral exposure matrices **X** = {*x*_*ij*_}_*n*×*n*_ and deliver different topological properties. Note that the total borrowing and lending may not be equal at a given interbank market rate. After the matching, the remaining funds to lend will be left in the account with the central bank. The rest funds banks want to borrow will trade with the central bank (outside the interbank network).

First, we use the closest-matching or minimum-distance algorithm to obtain a sparse network. Each bank’s total borrowing and lending are sorted separately in descending order. Then the transactions are assigned in that order (banks do not lend to themselves; in that case, the algorithm restarts with a random swap in the sorting of the banks). The largest lender *i* trades with the largest borrower *j*, and the final trading volume is min{*bl*_*i*_, *bb*_*j*_}. The banks whose demand for borrowing or supply for lending is already met will not be considered in the subsequent matching rounds. Banks with unmet demand or unmet supply will continue to trade with the banks whose remaining lending or borrowing is closest to theirs in the next round until demand or supply is met for all banks. This approach is similar to the minimum density method proposed by Anand et al. [34]. They both aim to obtain a matrix with the lowest network density, as the cost of linkages between banks can be costly.

Second, we use a random-matching algorithm with a loading factor to generate a different interbank network. In the algorithm, the counterparties are randomly matched. Specifically, the cells of the bilateral transactions in the matrix **X** = {*x*_*ij*_}_*n*×*n*_ are randomly selected and given by *x*_*ij*_ = *λ* min{*bl*_*i*_, *bb*_*j*_}. *λ* is a loading parameter used to control the network density. Usually, the smaller it is, the bigger the network density of the resulting random network is. The random matching algorithm captures the idea that interbank activity arises from stochastic liquidity shocks to banks [35]. Then, the interbank market structure will exhibit a random configuration.

Third, we further consider an ideal matching algorithm that can generate a network with minimum expected loss at interbank assets. It is solved in an optimization problem similar to Diem et al. [8]. Given the vectors **bl** and **bb**, the optimization procedure is to minimize the expected loss in the interbank exposure network by rearranging the linkages among banks. This matching is obtained technologically and does not represent the actual behaviors of banks. It serves as a reference for policy improvement. The network optimization problem is

$$\begin{array}{l} \underset{\left\{x_{ij}\geq 0,\forall i,j\in N\right\}}{\text{min}}\;\sum_{i}Loss_{i}^{interbank}=V\cdot \sum_{i}prob_{i}\cdot R_{i}\\ s.t.\left\{\begin{array}{l} \sum_{j\in N}x_{ij}=bl_{i}\\ \sum_{j\in N}x_{ji}=bb_{i}\\ x_{ii}=0,\forall i\in N\\ \sum_{j\in N}x_{ij}\cdot prob_{j}\leq \sum_{j\in N}x_{ij}^{t=0}\cdot prob_{j}\\ \sum_{i}\sum_{j}z_{ij}/(n^{2}-n)<a \end{array}\right. \end{array}$$ (7)

where *x*_*ij*_ represents lending from bank *i* to bank *j*, for ∀*i*, *j* ∈ *N*. *V* is the total economic value. *prob*_*i*_ is the expected default probability of node *i*. $x_{ij}^{t=0}$ denotes the original network’s lending. *R*_*i*_ measured with the DebtRank algorithm indicates the distress induced by node *i* in the system. ∑_*i*_∑_*j*_ *z*_*ij*_/(*n*^2^ − *n*) measures the network density where *z*_*ij*_ = 1, if *x*_*ij*_ > 0, and *z*_*ij*_ = 0, if *x*_*ij*_ = 0. *a* is the upper limit of network density given exogenously (We set it in the simulation to the value of the maximum network density in the initial network). Besides, the objective function is indefinite (non-convex problem), so we build a network simulation method to seek the solution. The solution process of the optimization problem (7) is detailed in **Appendix A in** S1 File.

In Eq (7), the first constraint is a row-sum constraint, i.e., it requires that the total amount borrowed by each bank remains unchanged. The second constraint is a column-sum constraint, i.e., it requires that the total amount lent by each bank remains unchanged. The third constraint requires that the diagonal elements in the matrix equal zero because banks do not trade with themselves. The fourth constraint is the counterparty credit risk constraint. Each bank’s average risk-weighted exposure should not be higher than before optimization. Moreover, we add a network density constraint (the fifth constraint) to the optimization problem to ensure the optimized networks are comparable with the minimum-density networks.

### 3.3 Price shocks to banks’ loan portfolios

A fall in the market price of loan assets can put pressure on banks and increase the financial vulnerability of the banking system. This shock to the fair value of banks’ loan portfolios could be driven by increased non-performing loans in the real economy. The shock propagation mechanism is referred to Bluhm [7] and Cifuentes et al. [36]. When banks cannot meet capital regulatory requirements, they will sell *sell*_*i*_ share of loan assets in exchange for *P* · *sell*_*i*_ units of liquid assets to improve their capitalization.


$$\gamma^{*}=\frac{bl_{i}+P\cdot \left(ml_{i}-sell_{i}\right)+c_{i}+P\cdot sell_{i}-bb_{i}-d_{i}}{\chi_{1}\cdot P\cdot \left(ml_{i}-sell_{i}\right)+\chi_{2}\cdot bl_{i}}$$ (8)

$$\Rightarrow sell_{i}=\text{min}(ml_{i},\frac{ml_{i}\cdot P\cdot \left(\chi_{1}\cdot \gamma^{⋆}-1\right)+bl_{i}\left(\chi_{2}\cdot \gamma^{*}-1\right)-c_{i}+d_{i}+bb_{i}}{\chi_{1}\cdot \gamma^{*}\cdot P})$$ (9)

The liquidation of the loan portfolio will lead to a further price fall and a new round of asset sales. The credit market thus has a downward-sloping aggregate supply curve. The supply and demand functions in the market determine the price of loans. The inverse demand function is defined as

$$\theta (P)=D^{-1}\;(Supply(P))=\text{exp}(-\beta \sum_{i}sell_{i}(P))$$ (10)

where *β* is a positive constant used to scale the price elasticity. Following Bluhm [7], we set *β* as −*Log*(0.8)/∑_*i*_*ml*^*i*^. When the financial system sells all loan assets, the price is 80% of the initial.

After selling all loan assets, banks who still unable to meet regulatory requirements will face bankruptcy, and the risk of bankruptcy will trigger cascading defaults through the interbank exposure network. The risk transmission in the interbank exposure network is calculated based on the DebtRank algorithm [37]. After experiencing losses on interbank assets, banks unable to meet capital requirements will further sell their loan assets at a discount. The risk will spread back and forth between the interbank market and the credit market. The liquidation ends when P* = *D*^−1^ (*Supply*(P*)) and no banks will default.

Here, the fall in market price (denoted by *P*) refers to all loans, not to the loans of some specific bank. In this regard, we consider the correlation of banks’ loan portfolios to be 1; that is, when one bank sells part of its portfolio, the rest banks will suffer from the same price shock of loan portfolios. This simplification of the model follows Cifuentes et al. [36], Bluhm and Krahnen [28], Aldasoro et al. [30], and Bluhm [7]. Also, in **Appendix B in** S1 File, we test the robustness of our model by considering the heterogeneity of correlations among banks in a specific bank-firm lending network.

### 3.4 Equilibrium definition

The competitive equilibrium of the banking network model is defined as: (I) Banks’ portfolio allocations on {*c*_*i*_, *bl*_*i*_, *bb*_*i*_, *ml*_*i*_} satisfy their profit maximization; (II) banks’ optimal counterparty choices determine the network structure; (IV) the price (P*) of the loan portfolio satisfies Φ(P*) = *D*^−1^ (*Supply*(P*)).

### 3.5 Risk transmission and measurement of systemich risk

The network-based method is widely used to identify financial networks’ risk contagion channels and capture the build-up of systemic risk [38–40]. It is different from the market-based measures, such as (delta) conditional value-at-risk (CoVaR/ΔCoVaR) [41], systemic expected shortfall (SES) [42], and conditional capital shortfall measure of systemic risk (SRISK) [43]. Market-based measures can quantify the risk spillover effects, but the risk contagion path of financial institutions cannot be described [44].

When the financial system receives a shock, the risk is transmitted through direct (via interbank lending) and indirect (via fire-sales spiral) contagion channels. Specifically, price shocks to banks’ loan portfolios, arising from the increase in non-performing loans in the real economy, will put pressure on banks. Banks will sell their loan assets at a discount to improve their capitalization. Other banks holding overlapping assets will be affected, facing a decline in the total value of assets and an increase in the likelihood of default. When the banks are insolvent, the default risk resulting from a bank’s failure will transmit to others through the interbank exposure network, further leading to fire sales of loan assets. These two risk transmission channels reinforce each other, creating a positive feedback mechanism that ultimately leads to cascading failure in the banking system.

At the moment *T* of model equilibrium, the loss at bank *i*’s loan assets is

$$Loss_{i}^{loans}=P(0)\cdot loans_{i}(0)-P^{*}(T)\cdot loans_{i}(T)+\sum_{t}^{T}P(t)\cdot sell_{i}(P(t))$$ (11)

*P*(*t*) is the liquidation price of the loan asset at time *t*. Following Poledna et al. [45] and Poledna and Thurner [24], the loss in bank *i*’s interbank assets is

$$Loss_{i}^{interbank}=V\cdot prob_{i}^{*}\cdot \underset{\approx 1,\;\text{if}\;prob_{j}^{*}\ll 1}{\underset{︸}{\prod_{j\neq i}^{n}(1-prob_{j}^{*})}}\cdot R_{i}\approx V\cdot prob_{i}^{*}\cdot R_{i}$$ (12)

For all banks,

$$\sum_{i}Loss_{i}^{interbank}=V\cdot \sum_{i}prob_{i}^{*}\cdot R_{i}$$ (13)

$prob_{i}^{*}$ denotes the probability of default after the exogenous shocks to loans. This approximation in *V* denotes the total economic value, namely, ∑_*l*_*bl*_*l*_. *R*_*i*_ measures the distress induced by bank *i*’s failure in the system, excluding the initial distress, based on the DebtRank approach [37].

$$R_{i}=\sum_{j\in N}v_{j}H_{j}(T)-\sum_{j\in N}v_{j}H_{j}(1)=\sum_{j\in N}v_{j}H_{j}(T)-v_{i}H_{i}(1)$$ (14)

where *v*_*j*_ = *bl*_*j*_/∑_*l*_*bl*_*l*_ denotes the relative economic value of node *j*. *H*_*i*_(*t*) denotes the cumulative relative loss of bank *i*’s equity. Eq (12) is certainly valid when $prob_{i}^{*}\ll 1$, or the individual DebtRank is low (*R*_*i*_ ≈ *v*_*i*_).

Following Roncoroni et al. [46], the systemic risk of the banking system is measured by

$$SR=\frac{\sum_{i}\left(Total\_Loss_{i}\right)}{\sum_{i}Total\_Assets_{i}}=\frac{\sum_{i}\left(Loss_{i}^{loans}+Loss_{i}^{interbank}\right)}{\sum_{i}\left(c_{i}+bl_{i}+ml_{i}\right)}$$ (15)

The systemic importance (systemic risk contribution) of bank *i* is defined as

$$SI_{i}=\frac{Loss_{i}^{loans}+Loss_{i}^{interbank}}{\sum_{i}\left(Loss_{i}^{loans}+Loss_{i}^{interbank}\right)}$$ (16)

The systemic vulnerability of bank *i* is

$$SV_{i}=\frac{Loss_{i}^{loans}+Loss_{i}^{interbank}}{e_{i}}$$ (17)

## 4. Optimization results

### 4.1 Data and parameters

We select 2010–2019 year-end balance sheet data for state-owned commercial banks, joint-stock commercial banks, urban commercial banks, and rural commercial banks as our sample. The sample excludes banks not yet established at the end of 2010 or closed at the end of 2019 (Data disclosures for unlisted banks in 2020 are incomplete). The top 50 banks in total interbank assets are selected as the final sample. According to data statistics, the total interbank assets (liabilities) of the selected top 50 banks account for 86.85% (90.65%) of the total interbank assets (liabilities) of the four types of commercial banks, indicating that the sample is representative. Missing data in 2010–2019 is filled in with the nearest non-missing values. The data on banks’ assets and liabilities come from the China Stock Market Accounting Research (CSMAR) Database and WIND Financial Database. Data processing and analysis are done in MATLAB (R2021b). Table 2 summarizes the parameters used in our model. Based on these ten years of data, we estimated our model separately and finally obtained ten sets of results.

### 4.2 Networks with the same “aggregate interconnectedness”

Based on the banking network model in Section 2 and the parameter setting in Table 2, we obtain the interbank networks and measure their corresponding risk performance. Here, we use the closest-matching and random-matching (the loading parameter is set at 0.8) algorithms to estimate the interbank bilateral matrices, obtaining the minimum density and random networks. In the risk measurement process, a thousand exogenous shocks (Ψ) are simulated to calculate the systemic risk level of its network.

**Fig 4:**
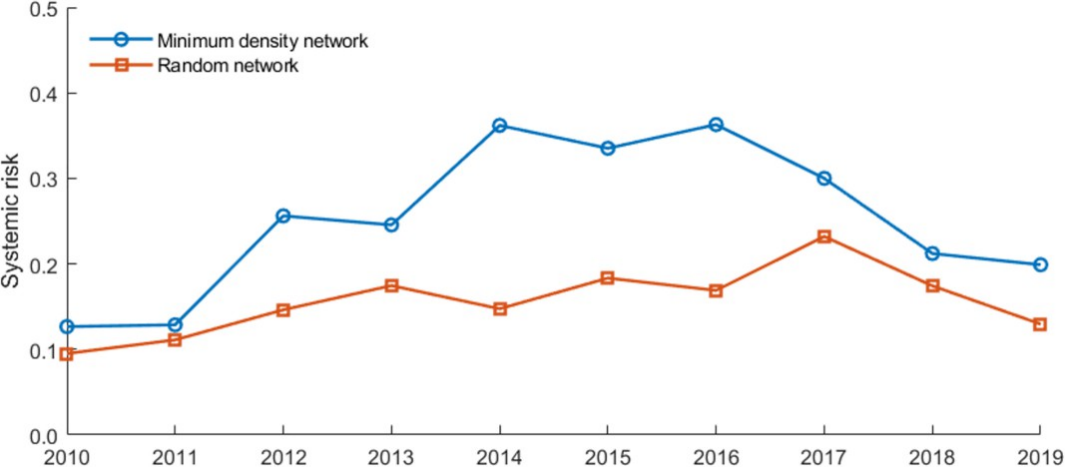
Overall systemic risk in banking networks with different matching algorithms.

Fig 4 shows the overall systemic risk in the different financial exposure networks. The results show that the overall systemic risk in the financial system with different matching algorithms differs significantly. In contrast, economic conditions, such as the interbank leverage, equity-liability ratios, and “aggregate interconnectedness (total interbank assets/liabilities, as shown in Table 3)”, do not change at a given point. It reveals that network configuration determined by banks’ bilateral trading patterns will affect the risk contagion mechanism in the networks, resulting in different losses.

**Table 3:** Annually average results of various networks.

	Minimum density network	Random network	Optimized network
Systemic risk	0.2528	0.1563***	0.0984***
Interbank funds	1743.8112	1743.8112	1743.8112
Total loans	8511.1809	8511.1809	8511.1809
Interbank leverage	1.3539	1.3539	1.3539
Equity-liability ratio	0.0873	0.0873	0.0873
Banks’ systemic risk contribution	0.0051	0.0031***	0.0020***
Banks’ interbank assets	23.3768	23.3764	23.3768
Banks’ interbank liability	23.3768	23.3764	23.3768
DebtRank of individual banks	0.6896	0.4033***	0.2030***

Note: Based on a two-sided paired T-test, *, **, and *** denote significance at 10%, 5%, and 1% levels, respectively. The results of the significance test of annual and individual differences of banks are summarized in the table, respectively.

**Fig 5:**
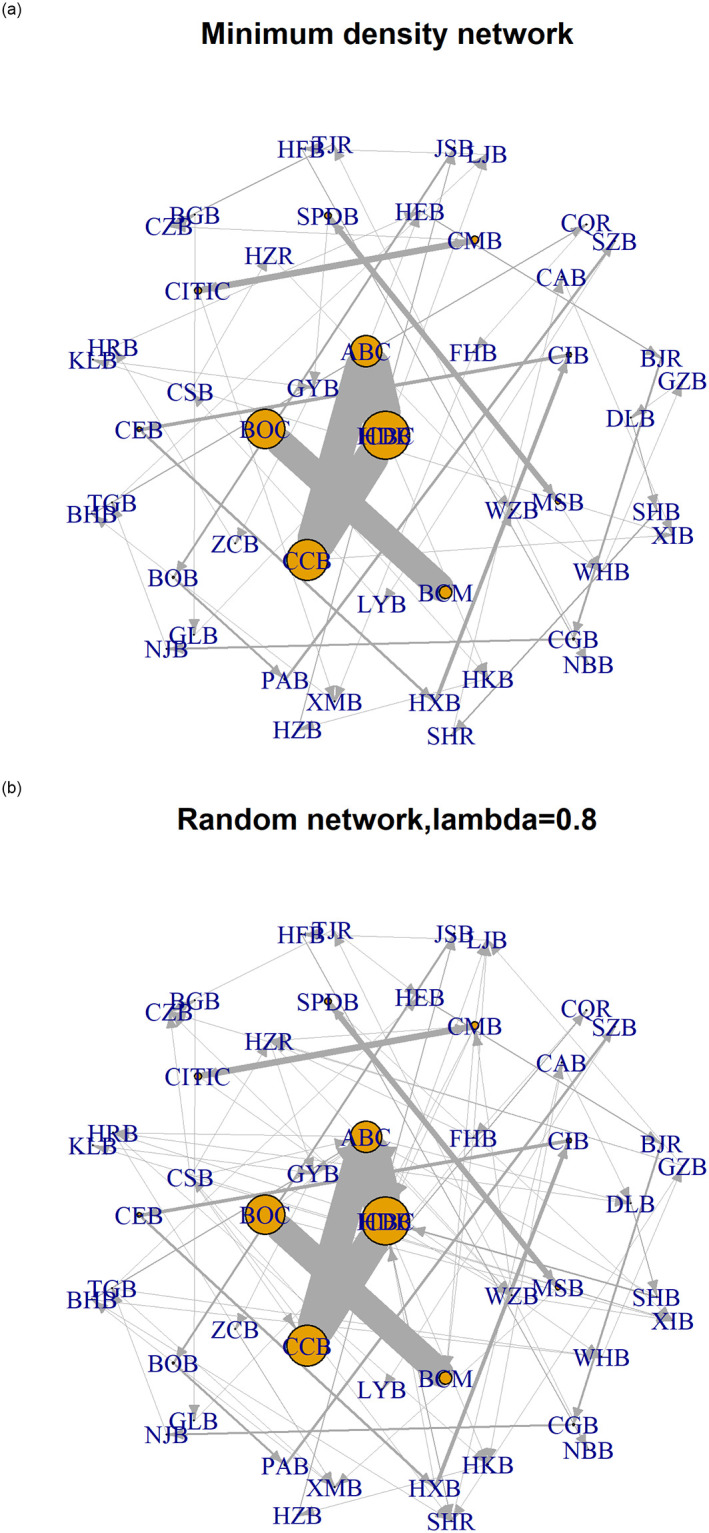
Interbank exposure network in 2010 with the closest matching and random matching. Bank equity determines the nodes’ size of the network, and the amount of interbank lending/borrowing determines the thickness of the gray lines linking the nodes. The node names are listed in **Table S1 of Appendix C in** S1 File.

Fig 5 presents the graphs of the interbank bilateral exposure networks in 2010 as an example. In Fig 5, the estimated interbank network shows a clear core-periphery structure, as identified by Craig and Von Peter [47], Fricke and Lux [48], and Covi et al. [49]. Large-scale/systemically-important banks are located at the network’s core, and others are located on the periphery. Furthermore, large-scale/systemically-important banks demonstrate apparent clustering in the minimum-density and random network.

### 4.3 Changes in network structures after restructuring networks

**Fig 6:**
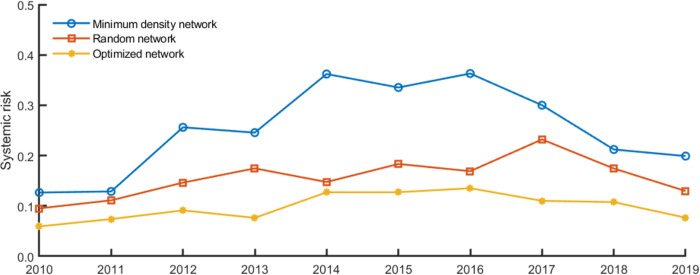
The overall systemic risk of the network before and after optimization.

This section uses the network optimization method to estimate the networks with minimal expected loss in the interbank network. Fig 6 compares the risk performance of the banking system before and after optimization. Table 3 summarizes the risk performance of different networks and presents the significance test results.

The results show that the systemic risk level is significantly lower for the optimized networks than for the minimum density and random networks. Compared with the minimum density network, restructuring network reduces the overall systemic risk by an average of 61.08% (= (0.2528−0.0984) / 0.2528) without worsening the initial economic conditions (banks’ capital buffer, total interbank assets/liabilities, and average risk-weighted exposure). The DebtRank is reduced by an average of 70.56% (= (0.6896−0.2030) / 0.6896) based on the optimization procedure of Diem et al. [8]. Diem et al. [8] may misestimate the potential for systemic risk reduction because they only consider the risk contagion in interbank networks.

**Fig 7:**
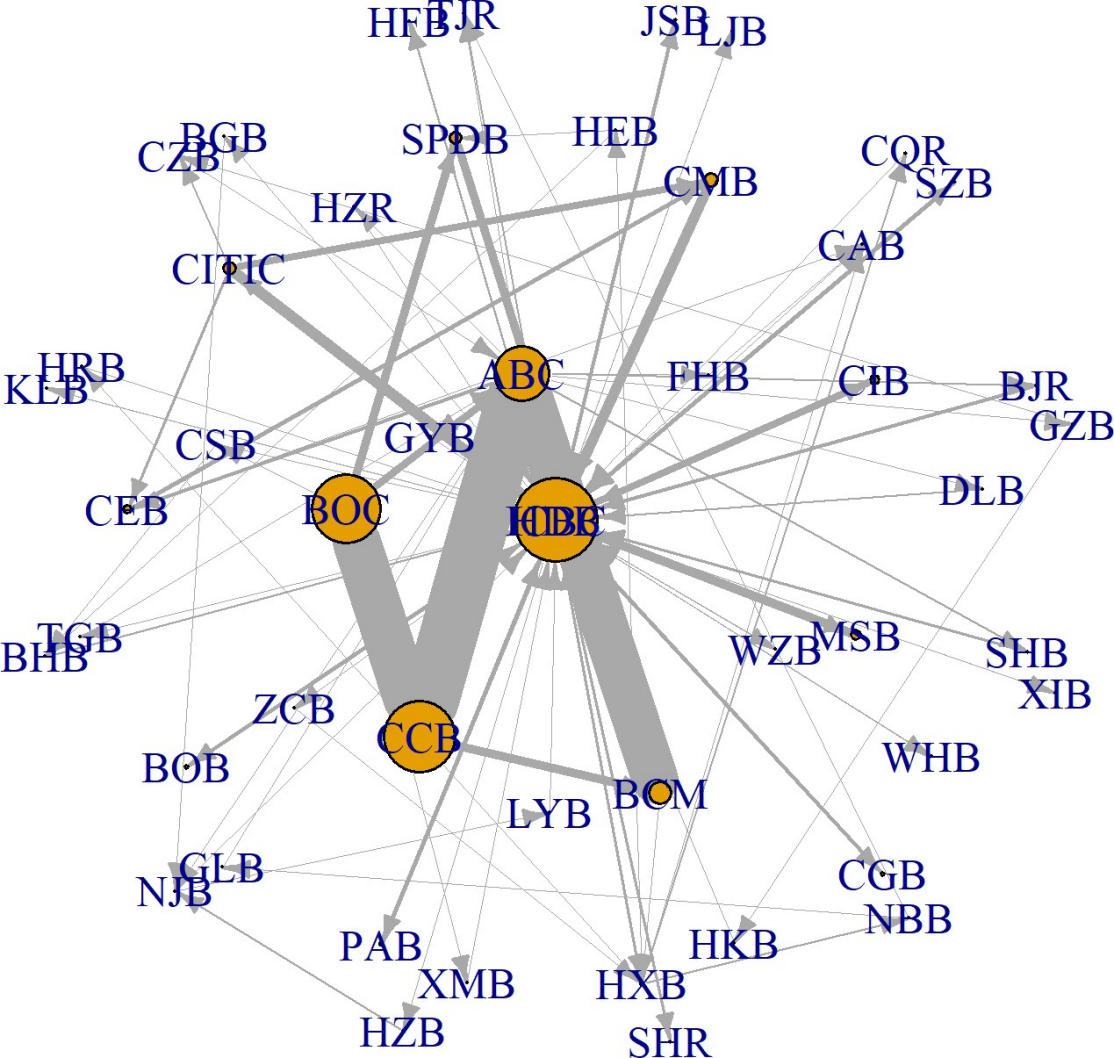
The optimized interbank network in 2010. The interbank networks before and after optimization of the other nine years are shown in **Fig S4 of Appendix D in** S1 File.

**Fig 8:**
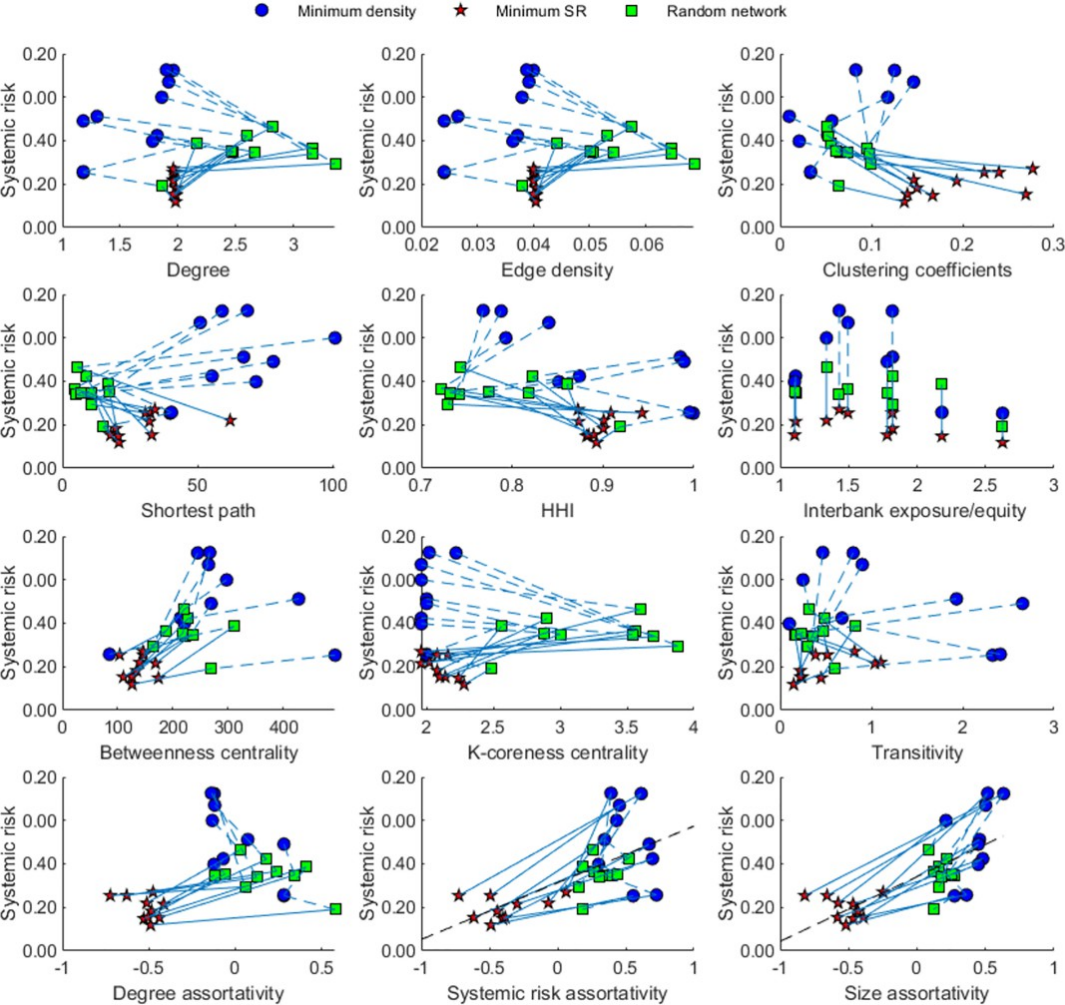
The relationship between systemic risk and network characteristics. The black lines in the figure are the fitted curves. Pearson’s linear correlation coefficients from left to right of the figure are 0.6391 and 0.7285, respectively (P values are all less than 0.01).

Fig 7 shows the topology graph of the optimized network. It reveals that the connections between the large commercial banks and small banks are more evident in the optimized network than in the minimum-density and random networks. To test if the change in the clustering pattern of large banks is the main reason for the systemic risk differences of the 30 networks, we compare various network characteristics of the 30 networks. Fig 8 shows the relationships between network characteristics and systemic risk. Table 4 summarizes the annual average values of topological measures and gives the results for the significance test.

**Table 4:** Annually average results of topological measures.

	Minimum density	Random network	Optimized network
Average degree	1.6080	2.5780***	1.9700**
Network density	0.0328	0.0526***	0.0402**
Clustering coefficients	0.0679	0.0982	0.1941***
Average shortest path	63.1401	10.7485***	30.3598***
Network concentration (HHI)	0.8883	0.8105***	0.8947
Ratio of interbank exposure to equity	1.6713	1.6711***	1.6713
Betweenness centrality	278.4240	228.9480	137.5720***
K-coreness centrality	2.0080	3.2100***	2.1020**
Transitivity	1.2524	0.3937**	0.5144*
Degree assortativity	0.0205	0.1284	−0.5201***
Assortativity mixing concerning banks’ systemic risk contribution	0.5183	0.2376***	−0.3849***
Size assortativity	0.4321	0.1044***	−0.5153***

Note: Based on a two-sided paired T-test, *, **, and *** denote significance at 10%, 5%, and 1% levels, respectively.

The node degree is the number of their neighbors. The network density is the ratio of the number of actual edges in the network to the number of all possible edges. The clustering coefficient describes the degree of aggregation between vertices in a graph, measured by the fraction of triangles around a node. The shortest path is from one node to another along the edges with a minimum sum of the weights on each constituent edge. Network concentration is measured by the Herfindahl-Hirschman Index (HHI), that is, ∑_*i*_∑_*k*_ (*x*_*ik*_/*bl*_*i*_)^2^. A value close to 1 indicates a high network concentration, and a value close to 0 indicates high diversification. The ratio of interbank lending to equity indicates the banks’ relative exposure to counterparties. The betweenness centrality measures a node’s importance in a network based on the shortest paths that pass through it. The k-coreness centrality measures the coreness of a node or group of nodes in a network based on the maximal subgraph in which all nodes have at least degree k. Transitivity is the ratio of “triangles to triplets” in the network.

We further use the measurement of assortativity coefficients defined by Newman [50, 51] and Leung and Chau [52] to describe the clustering pattern of similar banks in some aspects. Degree assortativity measures the similarity of connections in the graph concerning the node degree. The assortativity mixing concerning banks’ systemic risk contribution (*SR*_*i*_) indicates the banks’ tendency to connect with other banks with a similar systemic risk contribution. The size assortativity measures banks’ tendency to link to others of similar size. The measurements of those assortativity coefficients are detailed in **Equations (S1)–(S3) of Appendix E in** S1 File.

Fig 8 and Table 4 show that there are no monotonic relationships between the systemic risk and the topological measures, such as node degree, network density, clustering coefficient, shortest path, network concentration, the ratio of interbank exposure to equity, betweenness centrality, k-coreness centrality, transitivity, and degree assortativity coefficient. These findings are consistent with Nier et al. [27], Gai and Kapadia [53], and Glasserman and Young [54]. Interestingly, the assortativity mixing concerning the banks’ systemic risk contribution or size explains most of the differences in systemic risk in the 30 networks. A more disassortative network in terms of banks’ systemic importance or size seems more resistant to risk contagion than a more assortative network. In other words, the systemic risk of the network is higher if SIBs/large banks are closely connected than if they are not. Banks’ systemic importance is significantly proportional to their size (see **Figs S6–S7 of Appendix F in** S1 File), so these two conclusions are consistent. Besides, we use a larger simulation range to check the robustness of their relationships, as detailed in **Appendix G in** S1 File.

The results reveal that the level of systemic risk is well correlated with the clustering patterns of SIBs. We confirm that the findings of Diem et al. [8] and Krause et al. [23] are robust in a complex network with multiple risk transmission channels. Therefore, it may be an effective way to guide network optimization by reducing the connections among SIBs and directing SIBs to connect more with non-systemic small and medium-sized banks rather than other SIBs.

### 4.4 Potential explanation for less risk of the optimized networks

**Fig 9:**
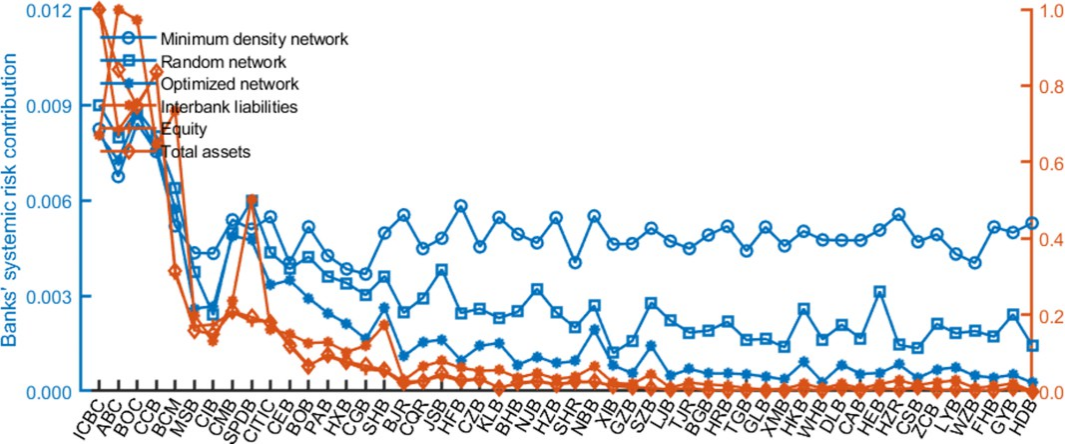
Banks’ systemic risk contribution before and after optimization. The risk contribution is averaged over ten years. The banks are in order by their total interbank assets. Figures below follow the same rules.

**Fig 10:**
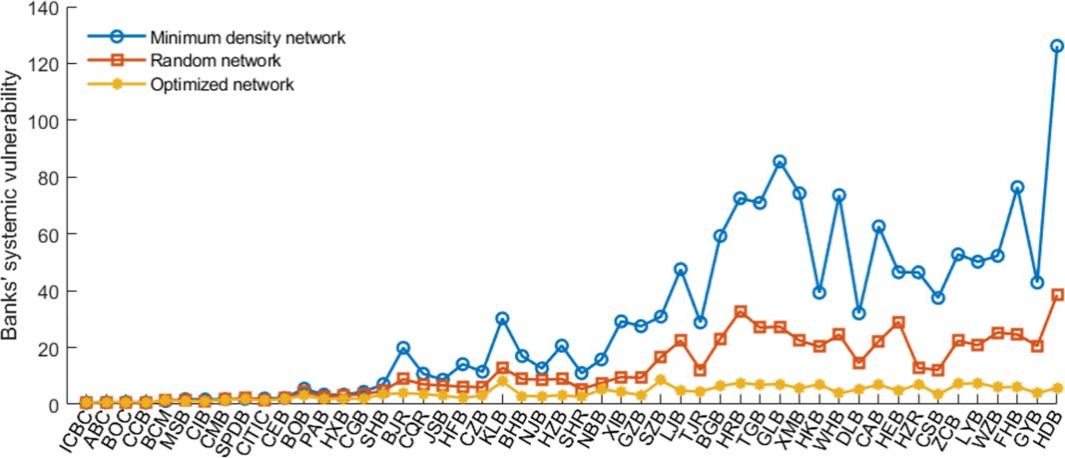
The banks’ average systemic vulnerability in different networks.

This section discusses the potential explanation for less risk of disassortative networks. Figs 9 and 10 report each bank’s systemic importance and vulnerability in different networks. We find that small and medium-sized banks’ systemic importance and vulnerability are significantly reduced in the optimized networks than in the minimum density and random networks. It indicates that a core with close connections will encourage systemic risk contagion, in line with Erol and Vohra [55]. In contrast, the close links among heterogeneous banks in the disassortative networks can enhance network stability because shocks to small and medium-sized banks are absorbed by larger banks that can bear more risk.

In addition, banks’ systemic risk contributions are proportional to their size (measured by their equity) and “aggregate interconnectedness” (measured by interbank liabilities) in networks, as shown in **Figs S6–S7 of Appendix F in** S1 File. It supports the importance of “too-big-to-fail” and “too-interconnected-to-fail” issues. Moreover, risk contributions are more proportional to size/ “aggregate interconnectedness” in optimized networks than in others. It means that network optimization tools help enhance the existing policies’ effectiveness.

## 5. Policy design

Above, the optimized network is a technical result rather than a real trading relationship. Based on the reference of the optimized network, this section proposes two policy tools to guide the networks to move toward the optimal design. Furthermore, we evaluate their effectiveness and compare them with the existing policy tools. It is worth noting that although limits on inter-SIBs exposure and pairwise capital requirements will have an impact on the importance of individual banks, they usually do not make SIBs to be non-SIBs. This is consistent with the current state of the Chinese banking system, as a bank’s systemic importance is highly dependent on its size and other factors, in addition to network connectedness. Moreover, even if that changes in a new round of (usually annual) ratings, the regulatory requirements are automatically met by the newly listed SIBs, according to the Chinese policy implementation logic. We do not need to identify SIBs internally and then implement policies.

### 5.1 Inter-SIBs exposure limits

First, we propose a tool to limit inter-SIBs exposure to reduce links among SIBs. This tool fundamentally differs from the large exposure limits set by the BCBS [56, 57] regarding a policy goal, but their implementation rule is quite similar. According to BCBS [56, 57] and Administrative Measures for Large Risk Exposures of Commercial Banks of China (2018), a single bilateral exposure of a bank cannot exceed 25% of its capital; for the exposure between two G-SIBs, the upper limit is 15%. Limiting interbank exposure aims to reduce the large bilateral exposure between individual banks and to guide network decentralization. Differently, our proposed inter-SIBs exposure limits aim to reorganize the bilateral trading relationships in different clusters and change the clustering pattern of SIBs.

Based on the list of G-SIBs published by the Financial Stability Board (FSB) in 2015–2020 and the conclusions in Fig 9, SIBs of interest in this paper include the Bank of China, the Industrial and Commercial Bank of China, the Agricultural Bank of China, and the China Construction Bank. This section first considers a limit for inter-SIBs exposure of 0.15; that is, bilateral exposure between two SIBs cannot exceed 15% of their capital. In other words, with limits only on inter-SIBs exposure, *limits*_*ij*_ = 0.15 · *e*_*i*_, if *i*, *j* ∈ *SIB*_*s*_, and *limits*_*ij*_ = inf, otherwise. *limits*_*ij*_ denotes the cap on interbank lending of bank *i* to bank *j*. Similarly, with large exposure limits set by the BCBS [56, 57], *limits*_*ij*_ = 0.15 · *e*_*i*_, if *i*, *j* ∈ *SIB*_*s*_, and *limits*_*ij*_ = 0.25 · *e*_*i*_, otherwise. With the limits on interbank exposure, the banks select the counterparties with the transaction amount closest to them. Then they trade the maximum amount allowed, that is, *x*_*ij*_ = min {*limits*_*ij*_, *bl*_*i*_, *bb*_*j*_}. After the lending of bank *i* to bank *j* reaches the upper limit, bank *j* will not be considered in the next round for bank *i*’s trading. After all rounds, if there still exists excess funds of bank *i*, it will be left in the account with the central bank (outside the interbank network), as in Batiz-Zuk et al. [58].

**Fig 11:**
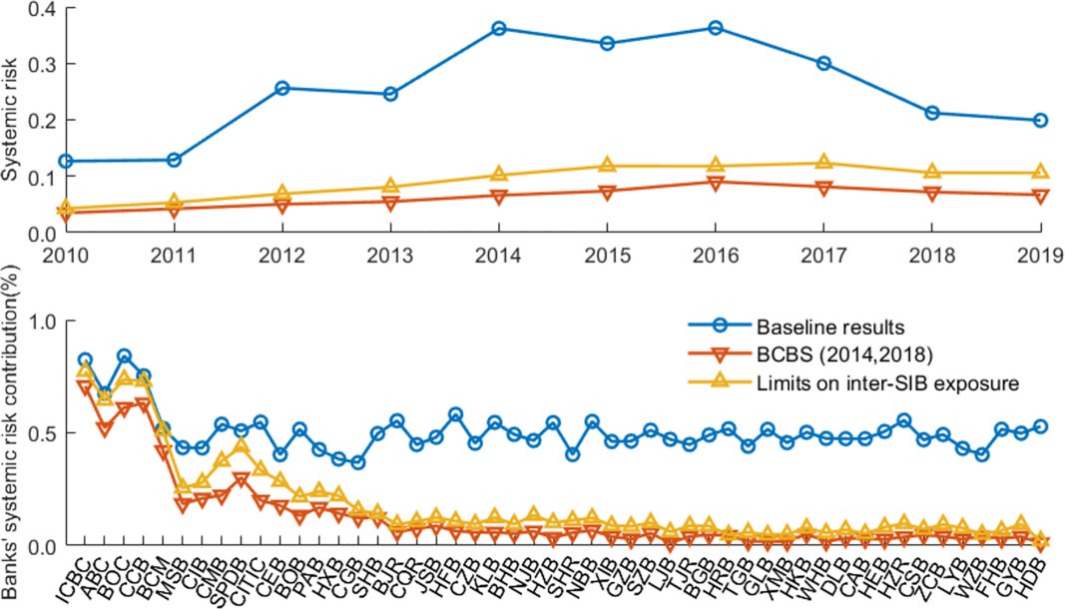
The systemic risk and banks’ risk contributions with limits on interbank exposure.

**Fig 12:**
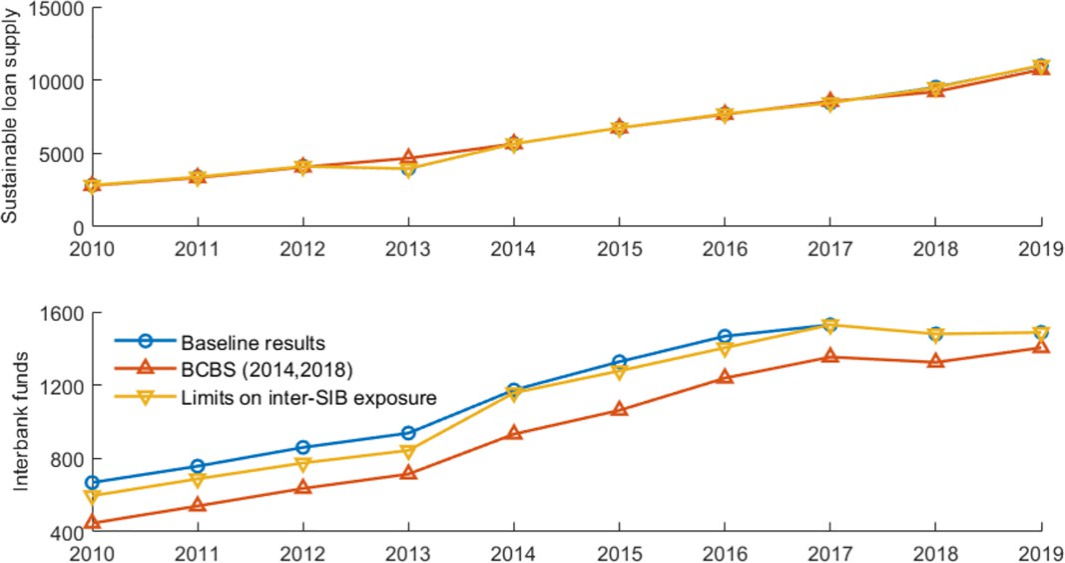
The sustainable loan supply and interbank funds with limits on interbank exposure.

Fig 11 presents the overall systemic risk and banks’ risk contributions in a banking network with limits on interbank exposure. Fig 12 illustrates the impact of policy tools on interbank market surplus and sustainable loan supply. We define interbank surplus as the sum of supply-side lending or demand-side borrowing at all banks. However, it is inadequate to consider only the interbank surplus. Social planners consider not only surpluses in the interbank market to set risk exposure but also those outside the interbank market, such as economic surplus. In this paper, we define market surplus (social welfare) as sustainable loans to the market (liquidity distributed to the real economy, measured by P*∑_*i*_*ml*_*i*_|Ψ) plus interbank market surplus (liquidity in the interbank market). Credit supply is a vital determinant of output [59]. Bluhm [7] also uses sustainable loan supply as a proxy variable for welfare. In this way, we can evaluate the effectiveness of policy tools in terms of systemic risk and social welfare. Table 5 summarizes the risk performance and network characteristics with limits on interbank exposure. These results are compared to the baseline results based on the closest-matching algorithm.

**Table 5:** Annually average results with interbank exposure limits.

	Baseline results	BCBS [56, 57]	Inter-SIBs exposure limits
Systemic risk	0.2528	0.0632***	0.0918***
Sustainable loan supply	6331.2610	6339.9654	6332.1072
Interbank funds	1168.8396	965.3742***	1123.8880***
Interbank leverage	1.3539	1.0746***	1.2808***
Equity-liability ratio	0.0873	0.0892***	0.0878***
Degree	1.6080	4.4760***	2.0400***
Density	0.0328	0.0913***	0.0416***
Degree assortativity	0.0205	−0.7561***	−0.6058***
Weighted degree assortativity	0.6047	−0.7657***	−0.5510***
Assortativity mixing concerning banks’ systemic risk contribution	0.5183	−0.8300***	−0.6608***
Network concentration (HHI)	0.8883	0.2868***	0.9112
Ratio of interbank exposure to equity	1.6713	0.8960***	1.6622***
Banks’ systemic importance	0.0051	0.0013***	0.0018***
Banks’ systemic vulnerability	29.5333	2.0710***	4.0555***

Note: Based on a two-sided paired T-test, *, **, and *** denote significance at 10%, 5%, and 1% levels, respectively. The results of the significance test of annual and individual differences of banks are summarized in the table, respectively.

Compared to the baseline results, the results show that the overall systemic risk level and banks’ average systemic importance/vulnerability significantly decrease when inter-SIBs exposure is limited, with a parameter of 0.15. The limits have no significant impact on the sustainable loan supply in the banking system; however, the limits cause a reduction in interbank market surplus. The potential reason is that small banks cannot withstand all the excess exposure of SIBs. In Table 5, with limits on inter-SIBs exposure, the assortativity mixing concerning banks’ contribution to systemic risk in the networks becomes negative, which means that this tool helps make the network more disassortative.

Furthermore, compared to the large exposure limits proposed by the BCBS [56, 57], the limits only on inter-SIBs exposure do not perform better at controlling systemic risk. However, our tool avoids the reduction in interbank liquidity to some extent, as shown in Table 5 and Fig 12. This finding is in line with expectations because the large exposure limits introduced by the BCBS [56, 57] have stricter requirements for bilateral transactions by non-SIB. In addition, the large exposure limits cause a significant increase in the node degree and network density, as shown in Table 5. If transaction costs are further considered, the large exposure limits might cause a further loss in interbank market liquidity.

With the large exposure limits proposed by the BCBS [56, 57], the assortativity coefficients mixing concerning banks’ systemic risk contribution and the network concentration reduce significantly. This finding also offers a new explanation for the effectiveness of the large exposure limits. Batiz-Zuk et al. [58] believe that when the excess exposure is left in the bank’s account with the central bank (outside the interbank exposure network), the risk of contagion will be reduced; when excess exposure is allocated to other counterparties, the risk of contagion in the financial network may increase due to the increase in interconnectedness. The findings in this paper extend this conclusion. If SIBs’ excess exposure is allocated to others, resulting in a more disassortative network with respect to banks’ systemic risk contribution, the stability of the network will also increase significantly.

**Fig 13:**
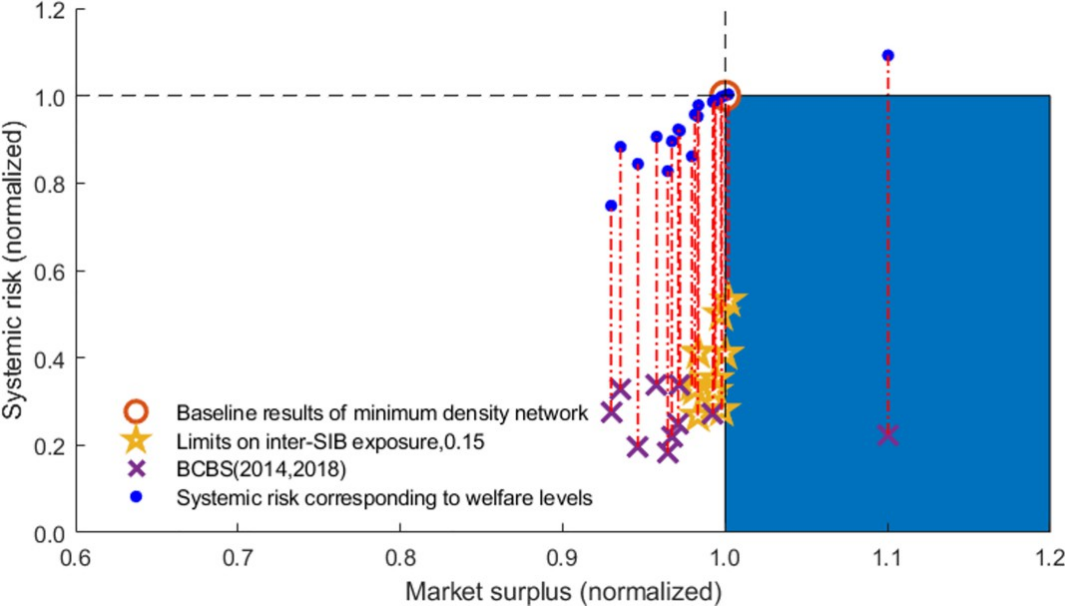
Effectiveness analysis of inter-SIBs exposure limits. The systemic risk and market surplus in the figure are normalized by dividing by the level of baseline results.

To distinguish whether the risk reduction comes from the decrease in interbank funds or the network arrangement, we further introduce the caps on aggregate interbank exposure. In Fig 13, the blue dots give the results of caps on aggregate exposure proposed by Coen and Coen [26] by changing the expectations of interbank funds in Eq (3). Also, results with caps on aggregate exposure are the control group, i.e., the linking relationships are unchanged, but the total transaction amount is reduced by a corresponding proportion. The red dotted lines indicate the changes in systemic risk level caused by the rearrangement of network linkages, excluding the decrease in market surplus. Table 6 gives the auxiliary statistical results with limits on interbank exposure to verify whether the changes in Fig 13 are significant.

**Table 6:** Auxiliary statistical results with limits on interbank exposure.

	Inter-SIBs exposure limits, 0.15	Cap on aggregate interbank exposure	Baseline results	BCBS [56, 57]
Systemic risk	0.0918	0.2287 ***	0.2528 ***	0.0632 ***
Social welfare	7455.9952	7455.9952	7500.1005***	7305.3396

Note: Based on a two-sided paired T-test, *, **, and *** denote significance at 10%, 5%, and 1% levels, respectively. The values in the table are annual averages.

Fig 13 and Table 6 show that the limits on inter-SIBs exposure effectively reduce systemic risk but worsen social welfare compared with baseline results. Furthermore, the red dotted lines support that the interbank exposure limits can hugely reduce systemic risk in the banking system by restructuring the network. In other words, the inter-SIBs exposure limits aim to improve market efficiency by rearranging the linking relationships among different clusters. Overall, the proposed limits on only inter-SIBs exposure offer regulators a reference in balancing economic growth and financial stability.

### 5.2 Pairwise capital requirements

The huge potential for reducing systemic risk by rearranging SIBs’ clustering pattern suggests that the contagion intensity within and between clusters is different. Based on this, we propose a tool for pairwise capital requirements. As shown in **Section 4**, the connections among SIBs tend to be risky, and the contagion intensity of links is greater within the SIBs’ cluster than outside it. Therefore, we impose a capital penalty on inter-SIBs transactions. The additional capital surcharges that bank *i* needs to hold are

$$\Delta \varsigma_{i}^{pairwise}=\text{min}(0.01\times \frac{\sum_{j\in N}weight_{ij}\cdot x_{ij}}{\text{min}(\sum_{j\in N}x_{ij})},0.025),i\in SIBs$$ (18)

where *x*_*ij*_ represents lending from bank *i* to bank *j*. Banks with higher weighted interconnectedness will be subject to higher capital requirements. Here, the weight between two SIBs is 1.5 times that of the other linkages, i.e., *weight*_*ij*_ = 1.5, if *i*, *j* ∈ *SIB*_*s*_, and *weight*_*ij*_ = 1, otherwise.

When SIBs have capital penalties due to trading with other SIBs, SIBs are assumed to prefer to exchange interbank funds with non-SIBs to shield themselves from the overly strict capital requirements. When non-SIBs are saturated, they then consider trading with other SIBs to meet their liquidity needs. Each trade follows the efficiency principle, and the amount traded between bank *i* and its selected counterparty bank j* is given by *x*_ij*_ = min(*bl*_*i*_, *bb*_j*_). The pairwise capital requirements aim to change banks’ trading patterns and, at the same time, impose higher capital penalties on banks that still trade with risky partners.

Pairwise capital requirements differ from the capital surcharges for systemically important banks (SIBs). According to the list of G-SIBs published by FSB and Administrative Measures for the Capital of Commercial Banks of China (2012), the Basel III capital surcharges for the four G-SIBs/D-SIBs in the Chinese market are expressed as

$$\Delta \varsigma_{i}^{Basel}=0.01,i\in SIBs$$ (19)

In fact, the Basel III capital surcharges for the G-SIBs/D-SIBs in China have changed recent years. For comparison purposes, we set the surcharge at 1% for all years.

Some literature also argues that differentiated capital can be levied based on eigenvector centrality [12], systemic risk contribution [60, 61], etc. Here, we analyze the effects of capital requirements based on banks’ contribution to overall systemic risk. The required capital buffer of bank *i* is set as

$$\Delta \varsigma_{i}^{SRi}=\frac{1}{e_{i}}\frac{SR_{i}}{\sum_{i}SR_{i}}\sum_{i}\left(\Delta \varsigma_{i}^{Basel}\cdot e_{i}\right),i\in SIBs$$ (20)

**Fig 14:**
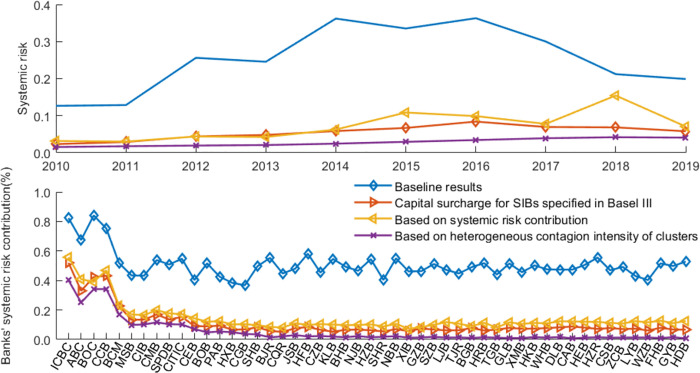
Overall systemic risk and banks’ systemic risk contribution with different capital requirements.

**Fig 15:**
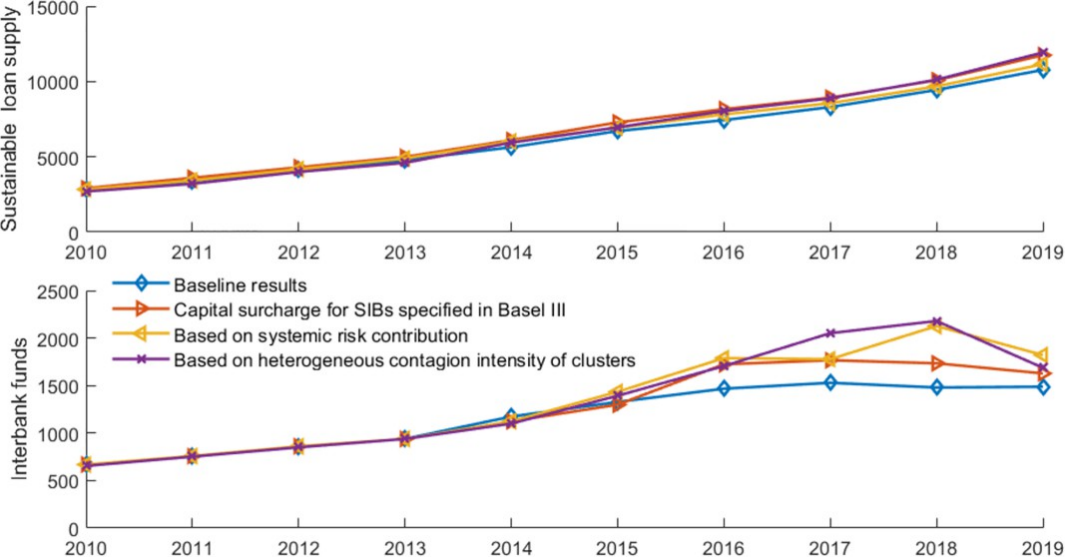
The sustainable loan supply and interbank funds with different capital requirements.

Fig 14 shows banks’ risk performance with different capital requirements. In the baseline results, interbank transactions follow the closest-matching rule and we use the parameters (except the capital buffer requirements) shown in Table 2. The results of the significance test and the average values are summarized in Table 7. Fig 15 shows the change in sustainable credit supply and interbank market surplus.

**Table 7:** Annually average results with the implementation of different capital requirements.

	Baseline results	Specified in Basel III	Based on systemic risk contribution	Based on heterogeneous contagion intensity
Systemic risk	0.2528	0.0550***	0.0722***	0.0284***
Sustainable loan supply	6343.1218	6810.5249***	6549.2408***	6635.9373*
Interbank funds	1168.8396	1249.8638*	1329.4315**	1330.9389*
Interbank leverage	1.3539	1.4163	1.4790**	1.4758*
Equity-liability ratio	0.0873	0.0867*	0.0861**	0.0861*
Degree	1.5320	1.6080	1.5300	1.9620**
Density	0.0313	0.0328	0.0312	0.0400**
Degree assortativity	0.0667	0.0205	0.0210	−0.5876***
Weighted degree assortativity	0.5774	0.6047	0.5706	−0.5096***
Assortativity mixing concerning banks’ systemic risk contribution	0.3907	0.5183**	0.3794	−0.6653***
Network concentration (HHI)	0.9098	0.8883	0.9036	0.9219
Ratio of interbank exposure to equity	1.6330	1.6713	1.6542	1.6563
Banks’ systemic importance	0.0051	0.0011***	0.0014***	0.0006***
Banks’ systemic vulnerability	29.5333	4.0452***	6.0730***	0.7577***

Note: Based on a two-sided paired T-test, *, **, and *** denote significance at 10%, 5%, and 1% levels, respectively. The results of the significance test of annual and individual differences of banks are summarized in the table, respectively.

The results show that overall systemic risk significantly declines with the implementation of the pairwise capital requirements, compared with the baseline results. Also, the assortativity coefficient becomes negative. It indicates that this proposed tool helps make the network more disassortative. Furthermore, pairwise capital requirements significantly affect the sustainable loan supply and interbank funds at the 10% significance level. In summary, the pairwise capital requirements based on the heterogenous contagion intensity of the bank cluster are an effective tool for reducing systemic risk without worsening social welfare.

Compared with the results for capital surcharges for SIBs specified in Basel III and the allocated capital surcharges for SIBs based on the systemic risk contribution (targeting the stability of individual SIBs), we find that our proposed pairwise capital requirements (targeting both the stability of individual SIBs and the clustering pattern of SIBs) perform better in reducing systemic risk. Basel III capital surcharges for SIBs and the reallocated capital requirements have no significant negative impact on the assortativity patterns of the network and fail to promote network optimization. Those findings highlight the importance of regulating the group interlinkages of SIBs, not only the risk performance of individual SIBs.

**Fig 16:**
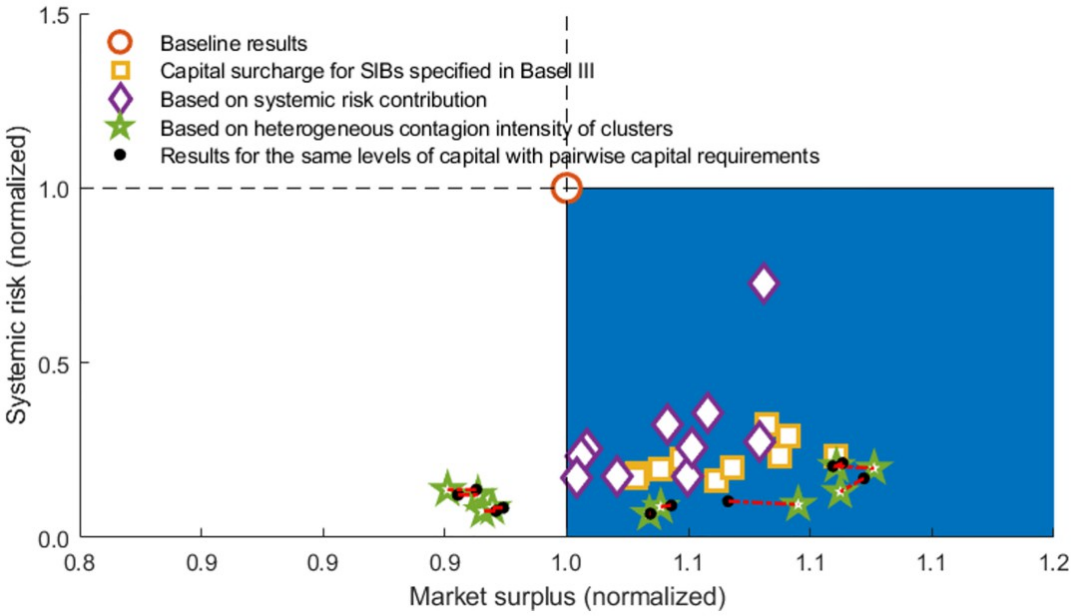
Effectiveness analysis of pairwise capital requirements. The systemic risk and market surplus in the figure are normalized by dividing by the level of baseline results.

To distinguish whether the risk reduction comes from the increase in capital requirements or the network arrangement, we further introduce a control group, as shown in Fig 16. The black dot denotes the results when banks are required to hold the same capital requirements level as pairwise capital requirements. However, they do not change their trading patterns, i.e., they still follow the closest-matching rules. The red dotted line indicates the reduction in risk due to the rearrangement of network linkages (SIBs prefer to trade with non-SIBS under the incentive of pairwise capital requirements), excluding the reduction due to a capital increase. Table 8 gives the auxiliary statistical results of different capital requirements.

**Table 8:** Auxiliary statistical results of different capital requirements.

	Based on heterogeneous contagion intensity	Baseline results	Specified in Basel III	Based on systemic risk contribution	Control group
Systemic risk	0.0284	0.2528***	0.0550***	0.0722***	0.0303*
Social welfare	7966.8762	7511.9613*	8060.3888	7878.6724	7946.5856

Note: Based on a two-sided paired T-test, *, **, and *** denote significance at 10%, 5%, and 1% levels, respectively. The values in the table are annual averages.

As shown in Table 8 and Fig 16, the proposed pairwise capital requirements can significantly reduce systemic risk without worsening the social welfare compared with the baseline case. Here, under the given shocks, the risk reduction described by the red dotted line is insignificant. With a broader range of exogenous shocks in Fig 18, the red dotted line further supports our findings that the optimization of network structure with the incentive of pairwise capital instruments is an essential reason for the decline in systemic risk. Our proposed tool helps to generate a more disassortative network structure, thus improving network stability. In addition, the pairwise capital instrument can be combined with the tools that limit inter-SIBs exposure to prevent banks from evading capital penalties through, for example, recapitalization.

### 5.3 Robustness analysis

**Fig 17:**
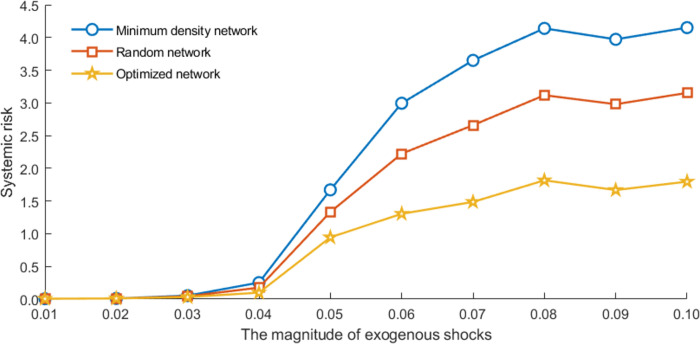
The results on different network topologies for changing the magnitude of exogenous shocks.

**Fig 18:**
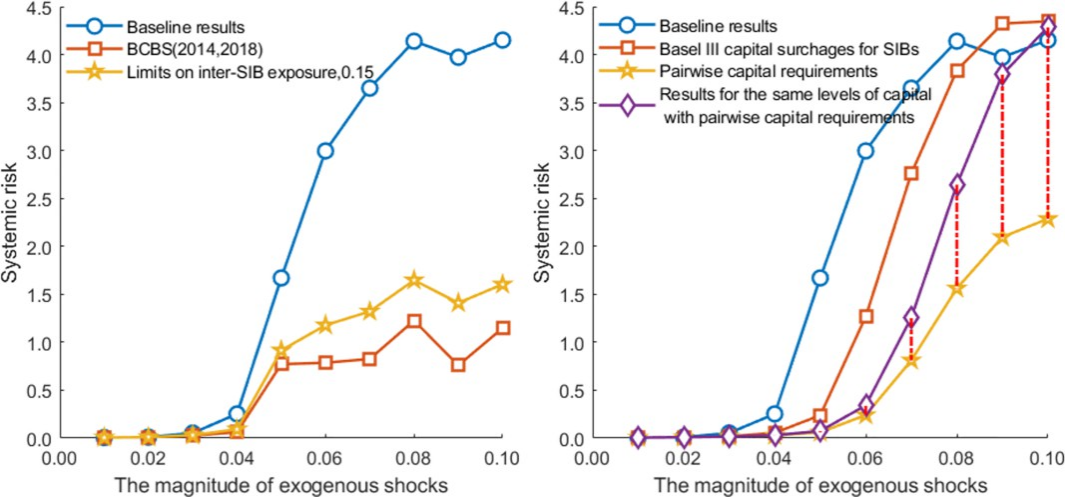
The results of the proposed policies for changing the magnitude of exogenous shocks.

In this section, we change the magnitude (*ms*) of exogenous shocks (Ψ ~ *N*(*ms*, 0.01))) to test the robustness of the above results, as shown in Figs 17 and 18. With the different magnitude of exogenous shocks, the better performance of the optimized network always exists, and the inter-SIBs exposure limits and pairwise capital requirements are still effective in reducing systemic risk. In other words, our findings are robust for different magnitudes of shocks.

Bardoscia et al. [62] introduce a modified DebtRank2. In general, DebtRank [37] is a lower bound to DebtRank2 [62]. Here, we test the robustness of our results by substituting DebtRank2 for the risk measure in interbank markets. Also, the results are in line with the above.

Furthermore, we test the robustness for the initial parameter setting of banks’ expected default probability (*prob*_*i*_) in two ways in **Appendix H in** S1 File. First, following Bluhm and Krahnen [28] and Bluhm [7], we endogenize *prob*_*i*_ in our model. This endogenous process fully accounts for banks’ capitalization, network interconnectedness, and other characteristics of individual banks. Second, we generate different values of *prob*_*i*_. We find that the model simplification of the expected default probability will not change our findings.

## 6. Conclusion

Based on the banking network model with a network optimization procedure, this paper explores the improvement of systemic risk prevention policies from the SIBs’ clustering pattern perspective. We have modified the network optimization method of Diem et al. [8] by including the reinforcement of the multiple risk transmission channels. The results show that the systemic risk contagion effects differ in networks with different connection tendencies of SIBs while the same “aggregate interconnectedness” remains. Rearranging interbank linkages without changing initial economic conditions could reduce system risk by about 61%. Specifically, the optimized networks exhibit a more disassortative pattern in terms of banks’ systemic importance than the minimum-density networks. Moreover, the systemic vulnerability and importance of small and medium-sized banks are significantly reduced in the optimized networks, which may account for the risk reduction. Thus, those findings offer a reference for the policy design that there is a great potential to mitigate risk contagion by reducing the business trading within the SIBs’ cluster and by forcing SIBs to trade with non-SIBs instead.

The proposed tools—inter-SIBs exposure limits and pairwise capital requirements—are useful in reducing systemic risk without worsening social welfare. Basel III capital surcharges for G-SIBs, which aim at enhancing the stability of individual SIBs, fail to optimize the network and are less effective in reducing the systemic risk than our proposed pairwise capital requirements, which take both the individual SIBs’ stability and their connection tendency into account. Capital/leverage requirements could be combined with network-based tools to ensure a substantive reduction in systemic risk.

This paper offers valuable suggestions for regulating network stability and perfecting systemic risk prevention policies. First, regulators should pay close attention to the impact of network structure changes described by SIBs’ clustering pattern on systemic risk contagion. Under exogenous shocks, different network structures will have different impact contagion effects. Regulators can establish early warning indicators of network-related risk contagion to guard against the systemic crisis. Besides, knowledge of the optimal network topology helps derive optimal benchmark networks for regulatory purposes. Second, regulators should not only strengthen network-based tools focusing on banks’ business interconnectedness but also note their coordination with the macro-prudential policy tools that have been implemented (such as the additional capital/leverage requirements for SIBs and the counter-cyclical capital requirements). Based on the theoretical model simulation results and the actual policy effect, regulators can formulate a flexible policy implementation mechanism at different stages of the financial cycle.

Several issues remain for future research. For example, this paper can be extended to study risk prevention and efficiency improvement in derivatives markets, bond markets, global markets, etc. In addition, it will be interesting for further research to focus on the cluster patterns of regional financial institutions in preventing systemic risk, which is related to the “too-many-to-fail” problems.

## Supporting information

S1 Data (RAR)

S1 File In this file, Appendix A discusses the solution process of the optimization problem; Appendix B shows the robust analysis on the correlations of banks’ loan portfolios; Appendix C introduces the bank list; Appendix D visualizes all the networks before and after optimization in 2010–2019; Appendix E measures the assortativity in networks; Appendix F reports the relationships between banks’ size and their systemic importance; Appendix G presents the robustness analysis on a larger simulation range for network optimization; Finally, the robustness analysis on the expected default probabilities is detailed in Appendix H. (DOCX)
