# Supplementary material for: Systemic risk prevention policies targeting systemically important banks: Does clustering pattern matter?
Source: PLoS One. 2023 Apr 27;18(4):e0284861. doi: 10.1371/journal.pone.0284861 (PMC10138215; doi:10.1371/journal.pone.0284861)
Supplement: S1 File — (DOCX) [file pone.0284861.s002.docx]

**Supporting information**

**Appendix A. The network optimization problem**

The measurement of systemic risk is calculated iteratively. Thus, we use the network simulation method with constraints to solve the optimization problem. The simulated networks can be obtained by solving the linear constrained optimization problem.

$$\begin{matrix} \min_{\text{\{}\mathbf{y}\text{\}}} \mathbf{P} \mathbf{y} \\ s.t.\left\{\begin{matrix} \mathbf{A}_{1}\mathbf{y}=\mathbf{C}_{1} \text{(I)} \\ \mathbf{A}_{2}\mathbf{y}=\mathbf{C}_{2} \text{(II)} \\ \mathbf{A}_{3}\mathbf{y}=\mathbf{0} \text{(III)} \\ \mathbf{A}_{4}\mathbf{y}=\mathbf{C}_{3} \text{(IV)} \\ \mathbf{y}\geq\mathbf{a} \text{(V)} \end{matrix}\right. \end{matrix}$$

where $\mathbf{P}$ is an $n^{2}$-dimensional vector whose elements are random variables (Extracting 10000 times and generating 10000 simulated networks based on a multivariate normal distribution). $\mathbf{y}=vec(\mathbf{X})$ is the “vectorization” of $\mathbf{X}=\{x_{ij}\}_{n\times n}$, namely $\mathbf{y}=\left(\begin{matrix} x_{11} & \begin{matrix} x_{21} & x_{31} & \cdots& \cdots\end{matrix} & x_{nn} \end{matrix}\right)_{1\times n^{2}}^{\top}$. It will not change the solution if we change the “equality (=)” to “less or equal than ($\leq$)” in the constraints (IV) of the optimization problem while the row and column sum are unchanged, see Diem et al. (2020).

Let $I_{n\times n}$ denote an ($n\times n$)-unit matrix; $\mathbf{1}_{1\times n}$ and $\mathbf{0}_{1\times n}$ represent the ($1\times n$)-matrices with all elements 1 and 0, respectively. $\mathbf{A}_{1}$, $\mathbf{A}_{2}$, $\mathbf{A}_{3}$, and $\mathbf{A}_{4}$ are the ($n\times n^{2}$)-matrices, specifically, $\mathbf{A}_{1}\text{=}\left(\begin{matrix} I_{n\times n} & I_{n\times n} & \cdots& I_{n\times n} \end{matrix}\right)_{n\times n^{2}}$,

$\mathbf{A}_{2}=\left(\begin{matrix} \mathbf{1}_{1\times n} & \mathbf{0}_{1\times n} & \cdots& \mathbf{0}_{1\times n} \\ \mathbf{0}_{1\times n} & \mathbf{1}_{1\times n} & \cdots& \mathbf{0}_{1\times n} \\ \vdots& \vdots& \ddots& \vdots\\ \mathbf{0}_{1\times n} & \mathbf{0}_{1\times n} & \cdots& \mathbf{1}_{1\times n} \end{matrix}\right)_{n\times n^{2}}$,

$\mathbf{A}_{3}\text{=}\left(\begin{matrix} \begin{matrix} 1 & & & \\ & 0 & & \\ & & \ddots& \\ & & & 0 \end{matrix} & \begin{matrix} 0 & & & \\ & 1 & & \\ & & \ddots& \\ & & & 0 \end{matrix} & \cdots& \begin{matrix} 0 & & & \\ & 0 & & \\ & & \ddots& \\ & & & 1 \end{matrix} \end{matrix}\right)_{n\times n^{2}}$,

and $\mathbf{A}_{4}\text{=}\left(\begin{matrix} I_{n\times n}\cdot prob_{1} & I_{n\times n}\cdot prob_{2} & \cdots& I_{n\times n}\cdot prob_{n} \end{matrix}\right)_{n\times n^{2}}$.

$\mathbf{C}_{1}$, $\mathbf{C}_{2}$, and $\mathbf{C}_{3}$ are the $n$-dimensional vectors

$\mathbf{C}_{1}=\left(\begin{matrix} bl_{1} \\ bl_{2} \\ \vdots\\ bl_{n} \end{matrix}\right)_{n\times1}$, $\mathbf{C}_{2}=\left(\begin{matrix} bb_{1} \\ bb_{2} \\ \vdots\\ bb_{n} \end{matrix}\right)_{n\times1}$, $\mathbf{C}_{3}=\left(\begin{matrix} \sum_{j} x_{1j}^{t=0}\cdot prob_{j}^{t=0} \\ \sum_{j} x_{2j}^{t=0}\cdot prob_{j}^{t=0} \\ \vdots\\ \sum_{j} x_{nj}^{t=0}\cdot prob_{j}^{t=0} \end{matrix}\right)_{n\times1}$.

The lower bound vector $\mathbf{a}$ is an $n^{2}$-dimensional vector: $\mathbf{a}=\left(\begin{matrix} a_{1} & a_{2} & \cdots& a_{n^{2}} \end{matrix}\right)_{1\times n^{2}}^{\top}$.

The linear program is to solve the vector $y=vec(X)$ with $n^{2}-n$ values unknown under the $3n$ linear equality constraints. We control the network density by controlling the non-zero number of the elements in the lower bound vector ($\mathbf{a}$). Thus, this approach can obtain various networks with different network densities. The global optimal solution to the non-convex problems (the objective function $V\cdot\sum_{i} prob_{i}\cdot R_{i}$ is indefinite) is still a puzzle. The optimization procedure is to find the optimal network with the possible minimum loss for a given method rather than the theoretically global optimum.

**Appendix B. Robust analysis on the correlations of banks’ loan portfolios**

Here, we consider the correlation arising from banks’ common exposure to the same borrower in a specific bank-firm loan network. Specifically, at time step *t*, we assume that a certain bank (bank *j*) goes bankrupt and liquidates all its loan portfolios ($x_{b^{j}f}$). This liquidation will push down the prices of such credit assets, thus affecting other banks. The price of the loan portfolio after shocks is

$$P_{f}(t\text{+}1)\text{=exp}(-\beta x_{b^{j}f}(P_{f}(t)))$$

As asset prices decline, bank assets depreciate. When the infected bank *j* fails to meet the capital requirement after asset depreciation, the value of the loan assets ($s_{i}(t+1)$) that bank *i* needs to liquidate is

$$\gamma^{*}=\frac{e_{i}(t+1)}{\chi_{1}\cdot(P\cdot ml_{i}-s_{i}(t+1))+\chi_{2}\cdot bl_{i}}$$

$$s_{i}(t+1)\text{=min(}\chi_{1}\cdot P\cdot ml_{i}(t+1)+\chi_{2}\cdot bl_{i}(t+1)-\frac{e_{i}(t+1)}{\gamma^{*}},ml_{i}(t+1))$$

When the bank fails to meet capital requirements but has no more loan assets to sell, we consider the bank to be insolvent. The insolvent bank is excluded from the banking system and no longer be considered in the subsequent contagion process. By referring to the practices of Greenwood et al. (2015), we assume that the banks’ target exposures remain fixed in percentage terms. So, all kinds of loan assets are sold simultaneously in proportion. Then the loan amount of bank *i* after the first round of liquidation is:

$$x_{b^{i}f}(t+1)=x_{b^{i}f}(t)-\frac{s_{i}(t+1)}{ml_{i}(t+1)}\cdot x_{b^{i}f}(t)$$

That is,

$$x_{b^{i}f}(t\text{+}1)=\left\{\begin{matrix} 0 (\mathrm{I}) \mathrm{bank} i \mathrm{is} \text{insolvent} \\ x_{b^{i}f}(t)\cdot\frac{ml_{i}(t+1)-s_{i}(t+1)}{ml_{i}(t+1)} (\mathrm{I}\text{I}) \gamma_{i}<\gamma^{*} \\ x_{b^{i}f}(t) (\mathrm{III}) \gamma_{i}>\gamma^{*} \end{matrix}\right.$$

(I) If bank *i* is insolvent before, its loan amount is zero; (II) If bank *i* is not insolvent before but fails to satisfy the capital requirement, it will liquidate part of the loan portfolios; (III) If bank *i* still meets the capital requirement, there is no need to liquidate any loan assets.

The liquidation of loan assets will further lead to the decline of loan asset prices. The anti-demand function of loan asset prices follows:

$P_{f}(t\text{+2})\text{=exp}(-\beta_{f}\sum_{k} sell_{b^{k}f}(P_{f}(t\text{+}1)))$, $k\supset i$, and $s_{b^{j}f}=x_{b^{j}f}$

where *j* is the insolvent bank exogenously given; $\beta_{f}$ is a positive constant to scale the price responsiveness with respect to loan assets sold; $sell_{b^{k}f}$ denotes the amount of loan assets *f* that bank *k* clears. Based on the selection principle of price elasticity ($\beta_{f}$) in Bluhm (2018), we set $\beta_{f}$ as $-Log\left(0.8\right)/\sum_{j} x_{b^{j}f}$, which means that the financial system sells all loan portfolios at 80% of the initial price.

After selling all loan assets, banks who still unable to meet regulatory requirements will face bankruptcy, and the risk of bankruptcy will trigger cascading defaults through the interbank exposure network. Then, the risk of bankruptcy will trigger cascading defaults through the interbank exposure network. After experiencing losses on interbank assets, banks unable to meet capital requirements will further sell their loan assets at a discount. The risk will spread back and forth between the interbank market and the credit market. The liquidation ends at time *T,* when $P_{f}^{*}=D^{-1} (Supply(P_{f}^{*}))$ and no banks will default. The cumulative loss of bank *i* at time *T* is:

$$loss_{i}(T)=\sum_{f} P_{f}(t)\cdot x_{b^{i}f}(t)-\sum_{f} P_{f}(T)\cdot x_{b^{i}f}(T)+\sum_{t+1}^{T} \sum_{k} sell_{b^{k}f}(P_{f}(t\text{+}1))$$

We use the 2010-2019 loan data between the 50 commercial banks and 1297 Chinese listed companies to build the bank-firm loan network. The data comes from the China Stock Market & Accounting Research (CSMAR) Database. Data preprocessing excludes loan data with the non-RMB settlement, unclear loan amount, vague bank name, or of non-traditional banks such as trusts. We unify the abbreviation and the full name of the same bank, and then the data with the same lending bank and the same borrowing company in the same year are merged.

According to the real bank-firm loan networks, we assume that the banks’ loan portfolios remain fixed in percentage terms in our banking network model. Then, we re-estimate our results, as shown in **Figures S1–S3**. Based on the new risk measurement method for the loss of a bank’s loan assets, we find that the changes in network characteristics after optimization and the effectiveness of inter-SIB exposure limits and pairwise capital requirements remain the same. In addition, our studies focus on interbank network optimization and the policy to guide the network to be disassortative. It will be very interesting to extend this paper to investigate the optimization of multi-layered financial networks and design policies from both ways in a new paper.

**Figure S1**. The relationship between systemic risk and network characteristics.

**Figure S2**. Effectiveness analysis of inter-SIBs exposure limits.

**Figure S3**. Effectiveness analysis of pairwise capital requirements.

**Appendix C. The list of banks**

**Table S1.** The list of 50 banks.

| Bank’s name in English | Abbreviation | Type |
| --- | --- | --- |
| Industrial and Commercial Bank of China | ICBC | State-Owned Commercial Banks |
| Agricultural Bank of China | ABC | State-Owned Commercial Banks |
| Bank of China | BOC | State-Owned Commercial Banks |
| China Construction Bank | CCB | State-Owned Commercial Banks |
| Bank of Communications | BCM | State-Owned Commercial Banks |
| China Minsheng Bank | MSB | Joint-Stock Commercial Banks |
| Industrial Bank | CIB | Joint-Stock Commercial Banks |
| China Merchants Bank | CMB | Joint-Stock Commercial Banks |
| Shanghai Pudong Development Bank | SPDB | Joint-Stock Commercial Banks |
| China CITIC Bank | CITIC | Joint-Stock Commercial Banks |
| China Everbright Bank | CEB | Joint-Stock Commercial Banks |
| Bank of Beijing | BOB | City Commercial Banks |
| Ping An Bank | PAB | Joint-Stock Commercial Banks |
| Hua Xia Bank | HXB | Joint-Stock Commercial Banks |
| China Guangfa Bank | CGB | Joint-Stock Commercial Banks |
| Bank of Shanghai | SHB | City Commercial Banks |
| Beijing Rural Commercial Bank | BJR | Rural Commercial Banks |
| Chongqing Rural Commercial Bank | CQR | Rural Commercial Banks |
| Bank of Jiangsu | JSB | City Commercial Banks |
| Hengfeng Bank | HFB | Joint-Stock Commercial Banks |
| China Zheshang Bank | CZB | Joint-Stock Commercial Banks |
| Bank of Kunlun | KLB | City Commercial Banks |
| China Bohai Bank | BHB | Joint-Stock Commercial Banks |
| Bank of Nanjing | NJB | City Commercial Banks |
| Bank of Hangzhou | HZB | City Commercial Banks |
| Shanghai Rural Commercial Bank | SHR | Rural Commercial Banks |
| Bank of Ningbo | NBB | City Commercial Banks |
| Xiamen International Bank | XIB | City Commercial Banks |
| Bank of Guangzhou | GZB | City Commercial Banks |
| Bank of Suzhou | SZB | City Commercial Banks |
| Longjiang Bank | LJB | City Commercial Banks |
| Tianjin Rural Commercial Bank | TJR | Rural Commercial Banks |
| Guangxi Beibu Gulf Bank | BGB | City Commercial Banks |
| China Resources Bank of Zhuhai | HRB | City Commercial Banks |
| Chongqing Three Gorges Bank | TGB | City Commercial Banks |
| Guilin Bank | GLB | City Commercial Banks |
| Xiamen Bank | XMB | City Commercial Banks |
| Hankou Bank | HKB | City Commercial Banks |
| Weihai City Commercial Bank | WHB | City Commercial Banks |
| Bank of Dalian | DLB | City Commercial Banks |
| Chang’an Bank | CAB | City Commercial Banks |
| Bank of Hebei | HEB | City Commercial Banks |
| Hangzhou United Rural Commercial Bank | HZR | Rural Commercial Banks |
| Bank of Changsha | CSB | City Commercial Banks |
| Zhejiang Chouzhou Commercial Bank | ZCB | City Commercial Banks |
| Bank of Luoyang | LYB | City Commercial Banks |
| Bank of Wenzhou | WZB | City Commercial Banks |
| Haixia Bank of Fujian | FHB | City Commercial Banks |
| Bank of Guiyang | GYB | City Commercial Banks |
| Bank of Handan | HDB | City Commercial Banks |

Note: The banks in the table are in order by their total interbank assets.

**Appendix D. Visualization of the network before and after optimization**

**Figure S4.** Interbank network topology graph before and after optimization in 2011-2019.

As shown in **Figure S4**, the connections between the large-scale/systemically-important banks appear to be fewer in the optimized network than in the minimum-density network.

**Appendix E. Measurement of assortativity in networks**

Newman (2002, 2003) defines the assortativity coefficients. The assortativity coefficient ($\overrightarrow{r}$) for mixing by vertex degree in a directed network is defined as

$\overrightarrow{r}=\frac{l^{-1}\sum_{(i,j)\in L} k_{i}^{\text{out}}k_{j}^{\text{in}}-\left[l^{-1}\sum_{(i,j)\in L} \frac{1}{2} \left(k_{i}^{\text{out}}+k_{j}^{\text{in}}\right)\right]^{2}}{l^{-1}\sum_{(i,j)\in L} \frac{1}{2}\left[\left(k_{i}^{\text{out}}\right)^{2}+\left(k_{j}^{\text{in}}\right)^{2}\right]-\left[l^{-1}\sum_{(i,j)\in L} \frac{1}{2}\left(k_{i}^{\text{out}}+k_{j}^{\text{in}}\right)\right]^{2} }$ (S1)

where $k_{j}^{\text{in}}$ and $k_{i}^{\text{out}}$ are the excess in-degree and out-degree of the vertices, respectively. $l$ is the number of edges. $L$ indicates the edge set.

The size assortativity coefficient is defined as

$r^{size}=\frac{l^{-1}\sum_{(i,j)\in L} Size_{i}Size_{j}-\left[l^{-1}\sum_{(i,j)\in L} \frac{1}{2}\left(Size_{i}+Size_{j}\right)\right]^{2}}{1^{-1}\sum_{(i,j)\in L^{2}} \frac{1}{2}\left(\left(Size_{i}\right)^{2}+\left(Size_{j}\right)^{2}\right)-\left[l^{-1}\sum_{(i,j)\in L} \frac{1}{2}\left(Size_{i}+Size_{j}\right)\right]^{2}}$ (S2)

Similarly, the assortativity mixing concerning banks’ systemic risk contribution is defined as

$r^{SR}=\frac{l^{-1}\sum_{(i,j)\in L} SR_{i}SR_{j}-\left[l^{-1}\sum_{(i,j)\in L} \frac{1}{2}\left(SR_{i}+SR_{j}\right)\right]^{2}}{l^{-1}\sum_{(i,j)\in L} \frac{1}{2}\left(SR_{i}^{2}+SR_{j}^{2}\right)-\left[l^{-1}\sum_{(i,j)\in L} \frac{1}{2}\left(SR_{i}+SR_{j}\right)\right]^{2}}$ (S3)

**Appendix F. Banks’ size and their systemic importance**

**Figures S6-S7** show the relationships between banks’ interbank liabilities/equity and their systemic importance. The results suggest the bank’s systemic importance relates to its interbank liabilities and equity. That is why regulators take the “too-big-to-fail” problem seriously. Also, some small banks have high levels of systemic importance. Furthermore, we use the Z value to test the significance of the three correlation coefficients. We find that banks’ systemic risk contribution becomes more proportional to their size and interbank liabilities in the optimized network than in others, with a statistical significance. The “too-interconnected-to-fail” problem in the optimized network might be mitigated because large banks remain systemic, and small banks do not have unnecessarily high levels of systemic importance.

**Figure S6**. Relationships between banks’ interbank liabilities and their systemic risk contribution for all ten years.

Note*:* The unit of banks’ interbank liabilities is 100 million.

**Figure S7**. Relationships between banks’ equity and their systemic risk contribution for all ten years.

Note*:* The unit of banks’ equity is 100 million.

**Appendix G. Robustness analysis on a larger range for network optimization**

This section conducts simulations with a larger range to test the robustness of the relationships between systemic risk and assortativity patterns of the network. For example, in the first-quarter data, we use the network simulation method with five constraints (as detailed in Appendix A) to generate a series of simulated networks. **Figure S5** shows the relationships between the loss of interbank assets (loss at bank *i*’s loan assets are assumed to be unchanged in this static optimization process) and assortativity coefficients. The results show that the level of systemic risk correlates well with the assortativity mixing concerning banks’ contribution to systemic risk. It suggests that the clustering patterns of SIBs are closely related to systemic risk.

**Figure S5.** The relationships between assortativity patterns of the network and loss in interbank assets.

Note: The minimum density network (initial network) is within our simulation range, indicating that our network simulation method has fully explored the sample space. The red lines are the fitted curves. The figure has some outliers that deviate from the trend, which might be affected by changes in other network characteristics.

**Appendix H. Robustness analysis on the expected default probabilities**

Here, we discuss the robustness of our findings in two ways. First, following Bluhm and Krahnen (2014) and Bluhm (2018), we can endogenize the parameters ($prob_{i}$) in our model. This endogenous process fully accounts for banks’ capitalization, network interconnectedness, and other characteristics of individual banks. Specifically, we simulate a series of price shocks to banks’ loan portfolios arising from the increase in non-performing loans in the real economy to assess the expected loss for each bank. The price shocks to loans are set high enough to mimic stress test scenarios and capture the effect of both indirect and direct risk channels. Next, we update the parameters ($EL_{i}=LGD\cdot prob_{i}$) and repeat the steps in Sections 3.1–3.3 until the financial system converges.[[1]](#footnote-1) After many simulations, we can approximate the distribution of the parameters and calculate the expected values of default probability ($prob_{i}$) and loss given default ($LGD$). Eventually, banks’ final equilibrium allocations on $\{c_{i},bl_{i},bb_{i},ml_{i}\}$ are determined.

The competitive equilibrium of the banking network model is defined as: (I) Banks’ portfolio allocations on $\{c_{i},bl_{i},bb_{i},ml_{i}\}$ satisfy their profit maximization; (II) the network structure is determined by banks’ optimal counterparty choices; (III) $r_{b}$ is the equilibrium market interest rate; (IV) the endogenous parameters $\{EL^{i}\}$ are parameters consistent with the equilibrium model; (V) the price ($P^{*}$) of the loan portfolio satisfies $\Phi(P^{*})=D^{-1} (Supply(P^{*}))$.

In the iterative process, the optimization procedure is dynamic. When the interbank linkages are rearranged, the probability of default might vary with the changes in risk contagion channels. When updating the probability of default, the banks’ interbank linkages will be arranged, too, because the fourth constraint in the optimization problem (7) will change.

**Figure S8.** The relationship between systemic risk and network characteristics.

**Figure S9.** Effectiveness analysis of inter-SIB exposure limits.

**Figure S10.** Effectiveness analysis of pairwise capital requirements.

After we include the endogenizing of parameter ($prob_{i}$) in our model, we re-estimate our main results, as shown in **Figures S8–S10**. The results are consistent with our findings.

Second, another way is to give the values of $prob_{i}$ exogenously based on empiricism, as Aldasoro et al. (2017) did. Considering the low expected probability of default for Chinese commercial banks, the results of our findings do not change when we give a priori the expected probability of Chinese commercial banks in the range of 0-1%, as shown in **Figure S11**. The results are consistent with our findings.

**Figure S11**. The robust test for the varying expected probability of default.

Furthermore, considering the expected probabilities of default for all banks are assumed the same, we further test the robustness when considering different expected probabilities of default for heterogeneous banks. Specifically, we assume $prob_{i}\text{\textasciitilde|N(0.005,0.003)|}$ to generate a suitable set of default probability values. The main results are shown in **Figures** **S12–S14**. We find that the results are also consistent with our findings.

**Figure S12.** The relationship between systemic risk and network characteristics.

**Figure S13.** Effectiveness analysis of inter-SIB exposure limits.

**Figure S14.** Effectiveness analysis of pairwise capital requirements.

**References**

Aldasoro, I., Gatti, D. D., & Faia, E. (2017). Bank networks: Contagion, systemic risk and prudential policy. *Journal of Economic Behavior & Organization*, *142*, 164–188.

Bluhm, M. (2018). Persistent liquidity shocks and interbank funding. *Journal of Financial Stability*, *36*, 246–262.

Bluhm, M., & Krahnen, J. P. (2014). Systemic risk in an interconnected banking system with endogenous asset markets. *Journal of Financial Stability*, *13*, 75–94.

Diem, C., Pichler, A., & Thurner, S. (2020). What is the minimal systemic risk in financial exposure networks? *Journal of Economic Dynamics and Control*, *116*, 103900.

Greenwood, R., Landier, A., & Thesmar, D. (2015). Vulnerable banks. *Journal of Financial Economics*, *115*(3), 471–485.

Newman, M. E. J. (2002). Assortative mixing in networks. *Physical Review Letters*, *89*(20), 208701.

Newman, M. E. J. (2003). Mixing patterns in networks. *Physical Review E*, *67*(2), 026126.

1. We consider that the financial system achieves convergence if the endogenous parameters do not change between two iterations, or if a financial system cycle is detected. The parameters enter a cyclical cycle when all banks in the financial system repeatedly choose the same allocations. [↑](#footnote-ref-1)
